# Impact of Combined Abiotic and Biotic Stresses on Plant Growth and Avenues for Crop Improvement by Exploiting Physio-morphological Traits

**DOI:** 10.3389/fpls.2017.00537

**Published:** 2017-04-18

**Authors:** Prachi Pandey, Vadivelmurugan Irulappan, Muthukumar V. Bagavathiannan, Muthappa Senthil-Kumar

**Affiliations:** ^1^National Institute of Plant Genome ResearchNew Delhi, India; ^2^Department of Soil and Crop Sciences, Texas A&M University, College StationTX, USA

**Keywords:** stress interaction, stress combinations, morpho-physiological traits, drought and pathogen infection, crop production, productivity

## Abstract

Global warming leads to the concurrence of a number of abiotic and biotic stresses, thus affecting agricultural productivity. Occurrence of abiotic stresses can alter plant–pest interactions by enhancing host plant susceptibility to pathogenic organisms, insects, and by reducing competitive ability with weeds. On the contrary, some pests may alter plant response to abiotic stress factors. Therefore, systematic studies are pivotal to understand the effect of concurrent abiotic and biotic stress conditions on crop productivity. However, to date, a collective database on the occurrence of various stress combinations in agriculturally prominent areas is not available. This review attempts to assemble published information on this topic, with a particular focus on the impact of combined drought and pathogen stresses on crop productivity. In doing so, this review highlights some agriculturally important morpho-physiological traits that can be utilized to identify genotypes with combined stress tolerance. In addition, this review outlines potential role of recent genomic tools in deciphering combined stress tolerance in plants. This review will, therefore, be helpful for agronomists and field pathologists in assessing the impact of the interactions between drought and plant-pathogens on crop performance. Further, the review will be helpful for physiologists and molecular biologists to design agronomically relevant strategies for the development of broad spectrum stress tolerant crops.

## Introduction

Due to global warming, and potential climate abnormalities associated with it, crops typically encounter an increased number of abiotic and biotic stress combinations, which severely affect their growth and yield (Mittler, 2006; Prasad et al., 2011; Atkinson et al., 2013; Narsai et al., 2013; Prasch and Sonnewald, 2013; Suzuki et al., 2014; Mahalingam, 2015; Pandey et al., 2015a; Ramegowda and Senthil-Kumar, 2015). Concurrent occurrence of abiotic stresses such as drought and heat has been shown to be more destructive to crop production than these stresses occurring separately at different crop growth stages (Mittler, 2006; Prasad et al., 2011). Abiotic stress conditions such as drought, high and low temperature and salinity are known to influence the occurrence and spread of pathogens, insects, and weeds (Coakley et al., 1999; Scherm and Coakley, 2003; McDonald et al., 2009; Ziska et al., 2010; Peters et al., 2014). They can also result in minor pests to become potential threats in future (Duveiller et al., 2007). These stress conditions also directly affect plant–pest interactions by altering plant physiology and defense responses (Scherm and Coakley, 2003). Additionally, abiotic stress conditions such as drought enhance competitive interactions of weeds on crops as several weeds exhibit enhanced water use efficiency than crops (Patterson, 1995; Ziska et al., 2010; Valerio et al., 2013).

The effect of combined stress factors on crops is not always additive, because the outcome is typically dictated by the nature of interactions between the stress factors (Atkinson et al., 2013; Prasch and Sonnewald, 2013; Pandey et al., 2015a,b; Choudhary et al., 2016; Ramu et al., 2016). Plants tailor their responses to combined stress factors and exhibit several unique responses, along with other common responses. Therefore, to fully recognize the impact of combined abiotic and biotic stresses on plants, it is important to understand the nature of such interactions. Mittler and colleagues developed a “stress matrix” to compile the interactions among various abiotic and biotic stresses on plant growth and productivity (Mittler, 2006; Suzuki et al., 2014). This matrix illustrates that the stress combinations can have negative as well as positive effects on plants. Therefore, development of plants with enhanced tolerance to combined abiotic and biotic stresses involves identification of physio-morphological traits that are affected by combined stresses.

Based on the currently available studies on the effect of concurrent stresses on plants, this review attempts to improve and amend the current understanding of stress combinations by explaining some fundamental concepts pertaining to them, highlighting their global occurrence and assessing their influence on crop growth. In this review, we provide a general overview of different stress combinations and their impact on agriculture and discuss in detail the effect of combined drought and pathogen infection on some important crops. The importance of undertaking simulation studies for assessing the impact of combined stresses on plants is also highlighted. Taking leads from some important studies on individual stresses, we have also presented some of the potential traits which can be utilized for crop improvement under combined drought and pathogen infection.

## Examples of Different Stress Combinations Occurring in Nature

Based on the number of interacting factors, stresses can be grouped into three categories: single, multiple individual, and combined stresses (Supplementary Figure S1). A single stress represents only one stress factor affecting plant growth and development, whereas multiple stress represents the impact of two or more stresses occurring at different time periods without any overlap (multiple individual) or occurring concurrently with at least some degree of overlap between them (combined). The co-occurrence of drought and heat stresses during summer is an example of a combined abiotic stress, whereas a bacterial and fungal pathogen attacking a plant at the same time represents a case of combined biotic stress. For example, brown apical necrosis of *Juglans regia* (walnut) is caused by combinations of fungal pathogens *Fusarium* spp., *Alternaria* spp., *Cladosporium* spp., *Colletotrichum* spp., and *Phomopsis* spp., and a bacterium, *Xanthomonas arboricola* (Belisario et al., 2002). A first stress factor preceded by another stress factor in sequence may either “endure” (due to priming) or “predispose” the plants to the subsequent stress. For example, drought predisposes *Sorghum bicolor* (sorghum) to *Macrophomina phaseolina* (causal agent of charcoal root rot) (Goudarzi et al., 2011). There are also scenarios where plants are exposed to “repetitive” stresses, where a single or multiple stresses are intervened by short or long recovery periods. For instance, incidences of multiple spells of hot days or multiple occurrences of drought and high temperature at different phenological stages of plants represent repetitive stresses.

Some examples of different stress combinations that are expected to arise due to climate change and their impact on plants is given in Supplementary Table S1. Simultaneously occurring drought and heat stress stands as the most evident stress combination (Prasad et al., 2011; Jedmowski et al., 2015). Likewise, plants growing in arid and semi-arid regions often face a combination of salinity and heat stress. High light stress also often accompanies heat stress. *Vitis vinifera* (grapes) growing in regions characterized by a continental climate, such as North China, face a combination of drought and cold stress which affects their productivity (Su et al., 2015). Plants growing in the Mediterranean region encounter combined cold and high light stress (Loreto and Bongi, 1989). *Triticum aestivum* (winter wheat) is also known to experience a combination of ozone and cold stress which reduces its frost hardiness (Barnes and Davison, 1988). Likewise, salinity combined with ozone stress reduces yields of *Cicer arietinum* (chickpea) and *Oryza sativa* (rice) (Welfare et al., 2002).

Similar to the different abiotic stress combinations, plants also encounter more than one biotic stresses simultaneously or sequentially. Infection by a combination of fungi, bacteria, and viruses are common and are known to cause severe disease symptoms, compared to infections by individual pathogens. Various biotic stress combinations and their impact on plants have been discussed by Lamichhane and Venturi (2015), and are also tabulated in Supplementary Table S1.

Plants also encounter biotic stressors simultaneously with abiotic stressors (Supplementary Table S2). The impact of environmental factors on plant diseases popularly known as the “disease triangle” has always been an important consideration for plant pathologists. Reports have documented the effect of drought or salinity leading to resistance or susceptibility of plants to *Puccinia* spp. (causal agent of rust), *Verticillium* spp. (causal agent of verticillium wilt), *Fusarium* spp. (causal agent of Fusarium wilt), *Pythium* spp. (causal agent of root rot), and *Erysiphe* spp. (causal agent of powdery mildew) (Supplementary Tables S1, S2). The influence of co-occurring drought (Valerio et al., 2013), high temperature (Cordes and Bauman, 1984), or cold (Patterson and Flint, 1979) stress on increased competitiveness of weeds over crops has also been documented.

## Stress Interactions as an Important Aspect Governing the Impact of Stress Combinations on Plants

**FIGURE 1:**
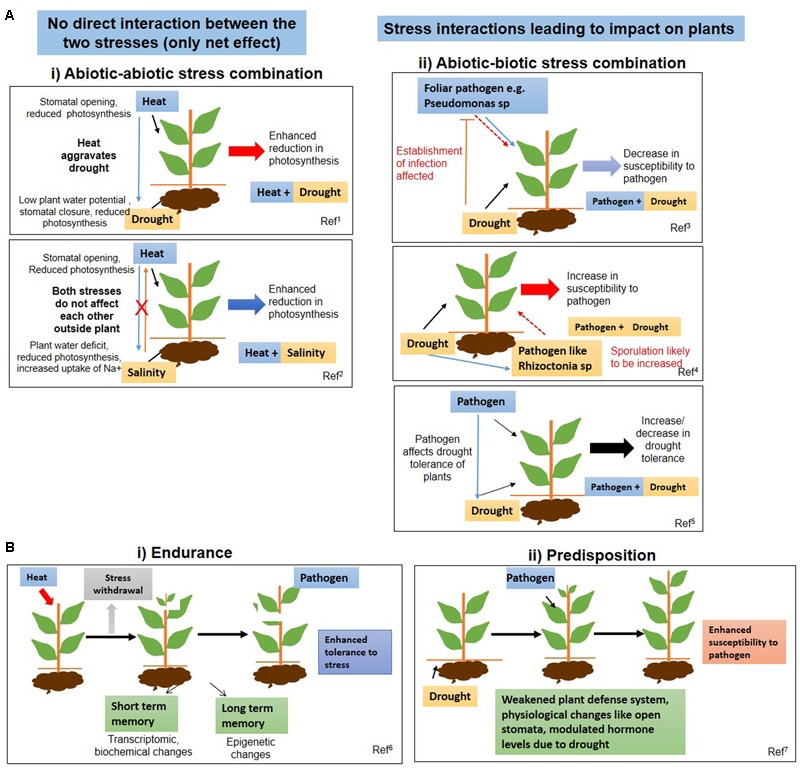
**Schematic representation of effect of stress combination on plants. (A)** Effect of combined stresses on plants is explained by representative examples of heat and drought (abiotic–abiotic stress) and drought and pathogen stress (abiotic–biotic stress) combination. **(i)** Depending on the nature of stresses, the two stresses can either not interact physically, but individually affect the plant leading to a net negative impact on plant growth or interact at plant interface and cause a net effect on the plant. Generally, abiotic stress combinations are examples of “only net effects and no stress interactions”. For example, simultaneous exposure to heat and salinity leads to enhanced retardation of physiological processes such as photosynthesis. **(ii)** Stress interactions are conspicuous in abiotic and biotic stress combinations wherein one stress factor affects the other stress factor *per se*. For example, exposure to combined drought and pathogen stress may result in a complex scenario encompassing an interaction of the two stresses along with the impact of the two stresses on the plant. Depending on the plant patho-system, the interaction may lead to enhanced or reduced susceptibility to a particular pathogen. Some pathogens also modulate drought tolerance of the plant. **(B)** Effect of multiple individual stresses (sequential stresses) on plants. Sequential stresses may either lead to priming or predisposition of plants to the subsequent stress as explained by examples of heat–pathogen and drought–pathogen stress combinations. **(i)** Priming: Exposure of plants to moderate heat stress (indicated by red arrow) may prime the plants to the subsequent pathogen infection. Mild stress can evoke stress memory in the form of epigenetic changes or transcriptomic changes in plants which may last short or long-term, leading to enhanced tolerance of stress to subsequent more severe stresses (same or different stress). **(ii)** Predisposition: A pre-occurring drought stress can pre-dispose plants to pathogen infection due to weakened plant defenses or any other metabolic changes occurring due to the drought stress. 1, Mittler, 2006; 2, Ahmed et al., 2013; 3, Gupta et al., 2016; 4, Sharma and Pande, 2013; 5, Xu et al., 2008; 6, Crisp et al., 2016; 7, Mayek-Perez et al., 2002.

Different types of stress interactions can have a range of effects on plants depending on the nature, severity, and duration of the stresses (**Figure 1**). In case of some abiotic–abiotic and majority of abiotic–biotic stress combinations, interactions not only occur between the plant and the stressors at the plant interface, but also directly between the stressors at or outside the plant interface (Supplementary Figure S2). In fact, the nature of such interactions between the stressors governs the magnitude of their impact on crop response. For example, a concurrent heat wave during a drought period may lead to more soil water evaporation resulting in aggravated drought conditions and increased crop yield loss. In addition to this, drought and heat stresses have synergistic effects on plant physiology, resulting in greater negative net impact manifested as drastic yield reductions (Mittler, 2006). Likewise, concurrent drought and weed stress further reduces water availability to crops and subsequently increases the competitiveness of weeds on them (Stuart et al., 1984).

In case of stress combinations involving heat and pathogen stress, high temperatures not only affect plants but also pathogens. Temperature is, in fact, one of the most important factors affecting the occurrence of bacterial diseases such as those caused by *Ralstonia solanacearum* (causal agent of wilt in tomato), *Acidovorax avenae* (causal agent of seedling blight and bacterial fruit blotch of cucurbits) and *Burkholderia glumae* (causal agent of bacterial panicle blight in rice) (Kudela, 2009). An increase in temperature modifies the growth rate and reproduction of pathogens (Ladanyi and Horvath, 2010). Temperature also affects the incidence of vector-borne diseases by altering the population development and spread of vectors. Similarly, the effect of salt stress on plant diseases might be the outcome of its modulation on the pathogen virulence, the host physiology and microbial activity in soils (Triky-Dotan et al., 2005). For example, increased incidence of *Fusarium* wilt in *Solanum lycopersicum* (tomato) under salt stress was found to be caused by more sporulation of the fungi under saline conditions (Daami-Remadi et al., 2009).

The combination of two stresses (abiotic–abiotic or abiotic–biotic) does not always lead to negative impact on plants. Some stress combinations negate the effect of each other, leading to a net neutral or positive impact on plants. One stress may also provide endurance to plants against another stress and hence yield is not always negatively impacted. For example, individual drought and ozone stresses are detrimental to the growth of *Medicago truncatula* (alfalfa), but the combination of drought and ozone results in increased tolerance of plants to the stress combination (Puckette et al., 2007). High CO_2_ has been shown to ameliorate the effect of drought stress in *T. aestivum* (Kaddour and Fuller, 2004) and *Poa pratensis* (bluegrass) (Song et al., 2014). Likewise, an increase in CO_2_ level from 350 to 675 ppm favored the competitiveness of the C_3_ crop *Glycine max* (soybean) over the C_4_ weed *Sorghum halepense* (johnsongrass) (Patterson, 1995). *S. lycopersicum* exposed to combined salinity and heat stress performs better than plants subjected to these stresses separately (Rivero et al., 2014). Ozone treatment also provides enhanced resistance to *Puccinia* spp. in *T. aestivum, Pseudomonas glycinea* (causal agent of bacterial blight) in *G. max* and *Erysiphe polygoni* in *Pisum sativum* (pea) (Supplementary Table S1).

Some stress combinations exhibit far more complex interactions and their effect on plants are variable. Heat–pathogen and drought–pathogen stress combinations are examples of such complex interactions. For example, with increased temperature, *T. aestivum* and *Avena sativa* (oats) become more susceptible to *Puccinia* spp., but some forage species such as *Cynodon dactylon* (Bermuda grass) become more resistant to rust disease (Coakley et al., 1999). Heat–pathogen and drought–pathogen interactions can be regarded as two agriculturally important stress combinations. The impact of combined heat and pathogen interaction on plants has been discussed by Pautasso et al. (2012) and Garrett et al. (2006). In the present review, we specifically focus on drought and pathogen stress combination as a case study and discuss it as a model for understanding the impact of abiotic and biotic stress combinations on plants.

## Drought–Pathogen Stress Combination: A Model for Understanding Combined Abiotic–Biotic Stresses

**FIGURE 2:**
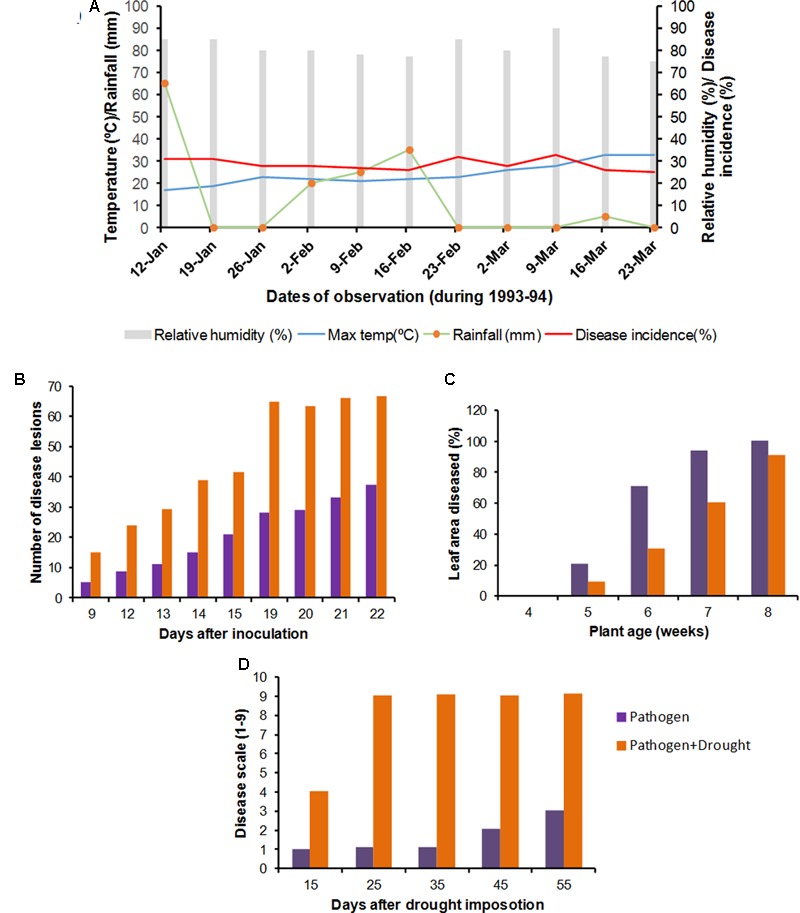
**Impact of combined abiotic stress and pathogen infection on plants.** The impact of combined abiotic stresses (mainly drought) and pathogen infection has been shown by taking examples from a few representative studies. **(A)** Impact of weather variables like temperature, rainfall and relative humidity (RH) on development of stem rot caused by *Sclerotinia sclerotiorum* in *Cicer arietinum* during the year 1993–1994 (Sharma et al., 2012). The figure shows increased incidence of stem rot under conditions of high humidity and high rainfall. **(B)** Effect of drought on *Puccinia recondita* infection in *T. aestivum*. Drought enhanced lesion development (Bethenod et al., 2001). **(C)** Effect of drought on *Erysiphe cruciferarum* infection in *Alliaria petiolata*. Drought and fungal infection had additive effect on plant growth. Drought although slowed disease development (decreased % diseased leaf area under drought conditions), plants under drought stress were much smaller as compared to well watered ones, so the powdery mildew occupied the total leaf area by the end of the experiment (Enright and Cipollini, 2007). **(D)** Effect of drought on infection by *Rhizoctonia bataticola* in *Cicer arietinum*. Drought (corresponding to 40% field capacity) predisposed chickpea to dry root rot (Sharma and Pande, 2013). All the graphs have been reconstructed from data taken from respective studies.

Drought stress interacts with pathogen infection both additively and antagonistically. On the basis of the number of reports of plant diseases being affected by drought stress and the frequency of occurrence of drought stress, this combination can be considered as one of the most important stress combinations affecting crop yields worldwide (**Figure 2**). Drought stress is reported to enhance the susceptibility of *S. bicolor, T. aestivum, Senecio vulgaris* (groundsel), *Hordeum vulgare* (barley), *Gossypium* spp. (cotton), and *C. arietinum* to *M. phaseolina, Puccinia* sp., *Erysiphe graminis* f. sp. *hordei, Fusarium oxysporum* f. sp. *vasinfectum*, and *Rhizoctonia bataticola*, respectively (Supplementary Table S1). On the other hand, drought stress is reported to provide endurance to tomato, *Medicago sativa* and *Arabidopsis thaliana* against *Botrytis cinerea* (causal agent of gray mold), *Oidium neolycopersici* (causal agent of powdery mildew), *Verticillium albo-atrum* (causal agent of verticillium wilt), and *Pseudomonas syringae* (causal agent of bacterial speck disease), respectively (Achuo et al., 2006; Gupta et al., 2016). In some cases, concurrent pathogen infection helps plants to endure drought stress, resulting in increased yield (Supplementary Figure S3) (Davis et al., 2014). For example, infection with *Cucumber mosaic virus* (CMV) led to improved drought tolerance of *Capsicum annum* (pepper), *S. lycopersicum*, and *Nicotiana tabacum* (tobacco) (Xu et al., 2008).

The effect of combined drought and pathogen infection at physiological and molecular levels has been discussed in a number of recent reports (Pandey et al., 2015a; Ramegowda and Senthil-Kumar, 2015; Choudhary et al., 2016; Gupta et al., 2016) and also summarized in Supplementary Figures S2, S3. In this review, we focus on some important plant diseases favored by drought stress.

One of the important diseases known to be aggravated by high temperature and water deficit conditions is dry root rot (DRR), caused by a necrotrophic fungus *R. bataticola*, Sharma and Pande (2013) have shown the interaction between *R. bataticola* and drought stress in laboratory conditions by infecting *C. arietinum* plants grown at different soil moisture contents with this fungi. This study showed that the disease incidence was the highest at 40% soil moisture content (**Figure 2D**). Less disease incidence at high soil moisture content was attributed to the inability of the fungal sclerotia to survive under wet soil conditions (Olaya and Abawi, 1996; Umamaheswari et al., 2000).

Long periods of drought accompanied with warm days and cool nights generally favor powdery mildew in *Beta vulgaris* (sugar beet) caused by the fungus *Erysiphe betae*. Increased occurrence of powdery mildew infection was observed in several parts of United States in the drought year of 1988 (Lamey, 1988). Occurrence of powdery mildew infections also coincided with extended periods of drought in Germany (Lamey, 1988). In contrast to the above report, drought stress delayed powdery mildew disease development in *Alliaria petiolata* (garlic mustard; Enright and Cipollini, 2007), which could have been due to osmotic stress mediated stomatal closure that typically reduces the pathogen’s ability to enter through the leaf (Thaler and Bostock, 2004). However, the exact reason for the same is not yet known. Enright and Cipollini (2007) showed that drought stress reduced plant growth, resulting in the powdery mildew fungi infecting all available leaf area by the end of the experiment, though there was a delay in disease development (**Figure 2C**). In cases such as this, although drought did not aggravate disease development, the net impact of the two stresses resulted in loss of plant performance.

Drought stress accompanied by high soil temperature has been correlated with increased charcoal stalk rot development, caused by *M. phaseolina*, in *S. bicolor* (Odvody and Dunkle, 1979; Mihail, 1989). This disease has also recently emerged as a threat in regions with warmer summers and low rainfall (Smith et al., 2015). Soil moisture content affects microsclerotia survival, root infection, and disease development. It has been found that microsclerotia can survive in dry soils for prolonged periods, but is unable to survive in saturated soils for more than a week (Mayek-Perez et al., 2002). Such interaction between drought and charcoal root rot has also been shown in *Phaseolus vulgaris* (common bean) under laboratory conditions (Mayek-Perez et al., 2002).

It has been reported that drought conditions in England and Wales have resulted in higher incidences of common scab caused by *Streptomyces scabiei* in *Solanum tuberosum* (potato) (Potato Council News, 2011). Infection occurs for 6 weeks after the start of tuber initiation and dry soils facilitate rapid infection of the fungus on developing tubers. The amount of scab on a tuber’s surface is directly related to the length of time that the plants are deprived of irrigation (Lapwood and Hering, 1968). The timing of drought occurrence also affects the severity of scabs on surface of tubers and it was found that drought during early stages of tuber development resulted in more scabs (Lapwood and Hering, 1968). Research by Davis et al. (1974) showed that irrigating fields to as high as 90% field capacity effectively suppresses common scab.

Given that a number of drought–pathogen stress combinations have a net negative influence on crop yields, it is important to devise strategies for improving crop performance under these stresses. A promising way of doing so is to identify measurable parameters or traits that are affected by combined stress conditions, which can be modified favorably to improve crop productivity under combined stress conditions. In the section below, we highlight some key traits that can be used for crop improvement under combined drought and pathogen infection.

## Potential Traits for Screening Genotypes for Tolerance to Combined Drought and Pathogen Infection

### Root System Architecture

Root system architecture (RSA) acts as a major interface between the plants and several biotic as well as abiotic factors and enables the plants to circumvent the environmental challenges by sensing and responding to them. The length and density of primary as well as lateral roots play a crucial role in drought stress tolerance. Development of high root length density (RLD) along with increased root diameter in response to drought stress confers drought tolerance in rice. For example, rice lines with low RLD show reduced drought tolerance (Allah et al., 2010). High RLD favors improved plant growth under drought conditions as it provides access to moisture present at deeper soil depths (Lynch et al., 2014; Zhan et al., 2015). Likewise, under drought stress, *Zea mays* (maize) with high RLD and few lateral roots had high plant water status, increased leaf photosynthesis, stomatal conductance, and increased overall growth, compared to plants with low RLD and more lateral roots. The presence of fewer but longer lateral roots results in enhanced rooting depth thereby increasing water acquisition from deeper layers of soil which helps in improved plant performance under drought (Lynch et al., 2014; Zhan et al., 2015).

Interestingly, RSA also plays a significant role under pathogen infection in plants. In an evaluation of virulence of *Pythium debaryanum* and *Pythium ultimum* (causal agents of root rot) on *T. aestivum*, plants with high root length had less fungal infection (Higginbotham et al., 2004). In contrast, infection of *Rhizoctonia solani* (causal agent of root rot) on *S. lycopersicum* caused reduction in total root length, number of root tips, and magnitude of root branching, which compromised water exploration from deep soil layers and consequently the shoot growth (Berta et al., 2005; Simonetta et al., 2007). Thus there seems to be a correlation between RLD and the extent of pathogen infection by root infecting fungi. Thus we speculate that increasing the RLD of plants might help in reducing pathogen infection.

Combined drought and root infecting pathogens cause greater damage to plants as both stresses appear to additively disrupt the RSA. For example, under drought conditions, *Fusarium solani* f. sp. *phaseoli* (causal agent of root rot in beans) infects the roots of *P. vulgaris* in deep layers of soil and affects water absorption. As a result of infection, accessibility to water present at deeper soil profiles is compromised under drought conditions, leading to severe reductions in plant growth (Dryden and Van Alfen, 1984).

Under combined stress, the time of occurrence of pathogen infection or drought stress has a significant effect on the net impact. *Phytophthora cryptogea* (causal agent of root and crown rot) infection on drought stressed *Carthamus tinctorius* (safflower) resulted in severe root rot disease development and a marked reduction in fresh weight of roots, compared to the conditions where drought stress followed the infection (Duniway, 1977). Similarly, *Phytophthora parasitica* (causal agent of root rot) infection on drought stressed *S. lycopersicum* resulted in greater disease severity, represented by an increase in the number of brown roots, reduced root length, and low fresh weight, compared to pathogen infection followed by drought stress (Ristaino and Duniway, 1989). Drought stress induced increase in root growth and exudation of amino acids (such as alanine and proline) and carbohydrates (such as pentose and glucose) are known to be responsible for enhanced root rot disease development on drought stressed plants (Schroth and Hildebrand, 1964; Duniway, 1977). Root exudates serve as nutrients for the growth of soil borne pathogens. These drought induced changes in the host physiology enhance pathogen infection by directly attracting more pathogens as well as intensifying the existing infection on plant roots. Additionally, pathogen infection modulates the composition of root exudates. Tomato roots infected with *F. oxysporum* f. sp. *radicis-lycopersici* exhibited decreased exudation of citric acid, but increased secretion of succinic acid as compared to the non-infected roots. Moreover, co-infection with bio-control bacterium *Pseudomonas fluorescens* WCS365 resulted in less disease and more secretion of succinic acid (Kamilova et al., 2006). Identification and analysis of exudates commonly secreted under drought and pathogen stress, and attempts toward manipulating the secretion of these exudates by inhibiting or over-expressing the secretory pathways may help in conferring tolerance to drought and pathogen infection. However, studies in this direction needs to be done to prove the suitability of this approach.

A number of studies have demonstrated no influence of drought stress on pathogen infection induced root damage in plants. For example, in an assessment of the effect of drought stress on infection by *Gaeumannomyces graminis* (Sacc.) var. *tritici* (causal agent of root rot) in wheat under low and severe drought stress conditions, Balota et al. (2005) found that infection under both the drought levels caused similar reduction in root dry mass. In addition, carbon assimilation rate and root decay were also found to be reduced similarly under both drought intensities, indicating that increasing drought intensities had little effect on disease development. Furthermore, *Pythium irregulare* and *R. solani* infection on *T. aestivum* cultivars under drought stress did not result in any significant change in root lesions inflicted by pathogen infection compared to infection on well-watered plants (Aldahadha, 2012).

Taken together, in most cases plant survival under concurrent drought and pathogen infection is compromised if RLD is affected as it influences the acquisition of water. Plants with the ability to maintain high RLD may perform better under combined drought and pathogen infection. Considering the role of RLD in both drought tolerance and pathogen infection, this trait can be utilized as a potential morpho-physiological trait for selecting cultivars with resistance to combined drought and pathogen stress. Additionally, root phenotyping tools can be exploited for screening plants with combined stress tolerance. Several studies have reported quantitative trait loci (QTLs) associated with RSA under drought stress (Comas et al., 2013). For example, a constitutive QTL, designated as root-abscisic acid 1 (ABA1), associated with root traits like branching, diameter, angle, and total dry mass has been identified in maize (Giuliani et al., 2005). Similarly, a QTL for ABA-induced reduction in lateral root growth and size has been identified in *A. thaliana* (Fitz Geral et al., 2006; Xiong et al., 2006). Moreover QTL (ARR2.1) for root rot resistance and tap root diameter (TD2-1) are correlated and increase in tap root diameter was related to enhanced resistance (Hagerty et al., 2015). Hence, we expect that some QTLs associated with efficient RSA may also be used in breeding programs to develop combined drought and pathogen resistant crops.

### Leaf Pubescence

Trichomes (leaf hairs) are modified epidermal cells found in uni- or multi-cellular, branched or unbranched, and glandular or non-glandular forms all over the surface of a plant. Though number and types of trichomes are genetically controlled, the environmental conditions also determine their pattern of occurrence. Plants grown in semi-arid environments maintain water levels by foliar absorption of water with the help of trichomes. Trichomes entrap water droplets and it has been shown that *Phlomis fruticosa* (Jerusalem sage) leaves with trichomes in mesophyll cells absorb dew deposits, which results in decreased water potential of drought stressed leaves, compared to the leaves of *Hedera helix* without trichomes. In addition, photosynthetic performance of hairy leaves is greater than that of non-hairy leaves under water stress conditions (Grammatikopoulos and Manetas, 1994). In some cases, drought conditions also increase trichome production in plants as a means of adaptation. For instance, drought stressed *Sinapis arvensis* (wild mustard) plants had more trichomes compared to control plants of the same line (Roy et al., 1999).

Studies have found that trichomes can serve as a barrier to infection by foliar pathogens (Lai et al., 2000). For example, *Phytophthora infestans* (causal agent of late blight) infection in *S. tuberosum* is negatively correlated with the presence of glandular trichomes. Presence of trichomes can reduce the relative humidity at the leaf surface, which is unfavorable for the germination of fungal spores (Lai et al., 2000). Trichomes may also secrete exudates that possess anti-fungal activities (Armstrong-Cho and Gossen, 2005; Nonomura et al., 2009). For example, exudates secreted by glandular trichomes present all over the plant surface of chickpea are shown to decrease infection by *Ascochyta rabiei* (causal agent of ascochyta blight) due to the anti-fungal properties of the exudates (Armstrong-Cho and Gossen, 2005). It was found that increased concentrations of exudates inhibited the conidial germination of *A. rabiei* while low concentrations promoted it. Identification of the pathways and genetic elements behind the glandular secretions from the trichomes under pathogen stress and their careful manipulation can enhance the resistance of plants.

In contrast to the above reports, trichomes in some plants may favor pathogen growth. For example, trichomes present on the leaf surface of common beans were reported to favor the growth of *P. syringae*. As trichomes retain water, exudates released from the broken cuticle at the base of trichomes might favor microbial growth (Monier and Lindow, 2003). Similarly, *A. thaliana* mutant *gl1* (GLABROUS1) plants with less trichome density were found to be tolerant to infection by necrotrophic fungus *B. cinerea* but *try* (TRYPTYCHON) mutants with more trichome density were found susceptible to infection (Calo et al., 2006). Not many studies have been done to probe into the role of trichomes in pathogen infection. A closer understanding of plant–pathogen interaction at this interface may help in further unraveling the role of trichomes in enhancing pathogen infection. It is evident that plants produce more trichomes under drought to minimize transpiration. In many cases, the presence of glandular trichomes has been shown to provide tolerance against pathogen invasion. However, as mentioned above, there are exceptions to this rule. Moreover, role of glandular trichomes and their secretory products under drought stress needs to be studied. Taken together, an extensive understanding of the nature of plant–pathogen interaction under drought stress would be needed in cases where trichomes enhance pathogen growth. Mapping for leaf pubescence related QTLs have been done for many plants like *Gossypium hirsutum* and *A. thaliana* (Lacape and Nguyen, 2005; Bloomer et al., 2014). It can be hypothesized that enhancement of trichome production impart protection against combined drought and pathogen infection in many cases and trichomes can be considered as a potential morpho-physiological trait conferring tolerance to the stress combination. Moreover, identification of QTLs related to leaf trichome density and secretion under drought and pathogen infection can also help in breeding genotypes better adapted to the combined stress. It can also be utilized for exploring the genes and pathways regulating trichome production and secretion which can be suitably modified to confer enhanced resistance under combined stress scenarios.

### Leaf Water Potential Regulation

A change in plant water potential is directly correlated to soil moisture level and is also affected by fungal and bacterial pathogens that disrupt the function of the plant vascular system. However, some traits related to maintenance of plant water potential are negatively affected by drought and pathogen stress. For example, plants close stomata under drought stress in order to reduce the transpirational loss of water. In contrast, infection by *Uromyces phaseoli* (causal agent of leaf rust) inhibits stomatal closure on *P. vulgaris* due to the toxins produced by the pathogen (Duniway and Durbin, 1971) which indicates that pathogen infection in cases like this can compromise drought tolerance.

Some pathogens may reduce plant water content even under sufficient soil moisture conditions. For example, *U. phaseoli* infection in *P. vulgaris* results in wilting at high soil water potential due to xylem damage, whereas uninfected plants experience wilting only under drought. Inhibition of stomatal closure by toxins secreted by *U. phaseoli*, disruption of cuticle layer and impaired stomatal resistance account for the increased water loss, which further reduce leaf water potential of plants under drought stress (Duniway and Durbin, 1971). Similarly, Burman and Lodha (1996) demonstrated a marked reduction in shoot water potential, leaf turgidity and transpiration in *Vigna unguiculata* (cowpea) plants subjected to concurrent drought and *M. phaseolina* (causal agent of charcoal rot and stem blight) infection. McElrone et al. (2003) showed that *V. vinifera* subjected to combined drought and *Xylella fastidiosa* (causal agent of leaf scorch) infection experience a significant reduction in leaf water potential and stomatal conductance, which aggravates the scorch symptoms more in drought stressed plants, compared to well-watered plants.

When *P. vulgaris* was exposed to simultaneous drought and *M. phaseolina* infection, high transpiration rate, decreased water potential and low stomatal resistance was observed in the stressed plants (Mayek-Perez et al., 2002). Drought stress caused plants to produce carbohydrates which facilitated the growth and infection of *M. phaseolina* (Mayek-Perez et al., 2002). In addition, it was found that varieties resistant to infection maintained high leaf water potential compared to susceptible varieties (Mayek-Perez et al., 2002). In case of charcoal rot due to infection by *M. phaseolina* in *G. max*, it has been found that maturation of the sclerotia was induced only by the reduced leaf water potential due to drought stress. It was also found that symptoms appeared only after imposition of drought stress. Likewise, Diourte et al. (1995) found that post flowering drought stress caused a reduction in leaf water potential in *S. bicolor*. Plants with reduced water potential had longer *M. phaseolina* lesions, which directly resulted in a reduction of grain yield. Pastor-Corrales and Abawi (1988) had demonstrated that drought-resistant bean varieties showed resistance to *M. phaseolina* infection as well.

Taken together, leaf water potential can be influenced by both drought and vascular pathogens and improved water status of plants under drought conditions might correspond to improved pathogen as well as drought resistance. One of the factors defining plants response to vascular pathogen infection is the xylem vessel dimension; *V. vinifera* genotypes with smaller xylem diameter are known to be less susceptible to infection by fungal vascular wilt pathogens (Pouzoulet et al., 2014). Identification of QTLs related to xylem diameter and xylem pit anatomy can be helpful to identify mechanisms for tolerance against combined drought and pathogen infection. Thus, plant water potential can be used as an important morpho-physiological trait to screen plants resistant to combined drought and pathogen infection.

### Cuticular Wax

Cuticle plays a vital role in protecting plants from drought stress and pathogen invasion. When stomata are closed under drought stress, a small amount of water is lost through cuticular layer. Cuticular layer also acts as a barrier to pathogen infection as it is hydrophobic and devoid of moisture (Martin, 1964).

The significance of cuticular layer has been studied under drought stress conditions. For example, drought stress led to an increase in the concentration of cuticular wax components such as alkanes, aldehydes, and ketones in *A. thaliana*, resulting in increased wax coverage in the stressed plants (Kosma et al., 2009). Under drought stress, the drought tolerant *T. aestivum* plants exhibit enhanced thickness of the cuticular layer while the susceptible varieties do not show any change in cuticle thickness (Hameed et al., 2002).

Likewise, the importance of cuticular wax has also been studied under pathogen infection. Marcell and Beattie (2002) exposed wild type and glossy mutants of *Z. mays* (*gl4*) to *Clavibacter michiganensis* (causal agent of leaf blight and Goss’s wilt of maize). Compared to the wild-type, more bacterial colonies were observed on the *gl4* mutants, which had a thin cuticular layer due to an alteration in the wax biosynthesis pathway (Marcell and Beattie, 2002). Nutrient and water exudation through the weak cuticular layer might have encouraged the colonization of bacteria, leading to more pathogen growth in the *gl4* mutants. Jenks et al. (1994) showed that bloomless (*bm*) mutants of *S. bicolor*, deficient in the synthesis of epicuticular wax and having a thin cuticular layer, were highly susceptible to infection by *Exserohilum turcicum* (causal agent of leaf blight) compared to the wild type plants. The rate of water loss was found to be high in the *bm* mutant compared to wild type plants. This apparently suggests that the thickness of cuticular wax can be used as a trait to identify plants tolerant to *E. turcicum*. Plants without stomata and deficient in cuticular wax have been used to study the significance of cuticular wax under pathogen infection. Isaacson et al. (2009) showed that penetration of pathogen through stomata was more with astomatous fruits (cutin deficient, *cd* mutant) of *S. lycopersicum*. Only the *cd* fruits were found to be infected with *B. cinerea* (causal agent of gray mold) depicting a role of cutin in pathogen resistance. Along with the cutin content, the composition and architecture of the wax layer also determines their role in defense. In the above study, it was also found that among the three cd mutants, cd1 which showed lack of microfissures, elevated level of amyrins and decreased levels of alkanes of chain length >30 showed maximum water loss and minimum susceptibility to *B. cinerea*.

Although, there are no studies showing the direct role of cuticular wax under combined drought and pathogen infection, the above evidence suggests its probable role in combined stress. Thus, plants produce more complex and thick cuticular wax layer in response to drought stress, which in turn might impart tolerance to pathogen infection. Additionally, the composition and architecture of the wax layer is equally important in defining the role of cuticle in defense mechanism. Detailed investigation of the pathways that determine the composition and structure of cuticle layer may help in identifying targets which can be manipulated to impart improved resistance to plants against combined drought and pathogen infection. Srinivasan et al. (2008) have found that QTLs for epicuticular wax, rate of water loss from excised leaves and harvest index co-located with QTLs associated with shoot and root-related drought resistance traits in rice. One example of such QTL is a region on chromosome 8 of rice. Considering the importance of cuticular wax in providing resistance against many pathogen infection, identification of QTLs linked to wax content and disease resistance should also be done. Cuticular wax may be considered as a trait that can be used to screen plants tolerant to combined drought and pathogen infection. The measurement of wax content can be made by simple weight analysis by immersing leaves in chloroform and determining the wax content after chloroform evaporation (Zhou et al., 2013). Thus, the trait can be efficiently utilized for large scale screenings of plants better adapted to combined drought and pathogen infection.

### Canopy Temperature

Canopy temperature (Tc) has been used to measure drought stress tolerance of plants (Gonzalez-Dugo et al., 2005). Tc varies with each leaf under drought and pathogen infection, as stress induced drooping and curling of leaf reflects radiation differently (Jackson, 1986). Tc plays a significant role in plant growth under drought stress. *T. aestivum* plants under drought stress were found to have high Tc and yielded less than irrigated plants (Blum et al., 1989). Plants which maintain low Tc under drought stress conditions possess high plant water status and thus are better adapted for drought stress (Blum, 2009).

The importance of Tc under pathogen infection has also been shown by Eyal and Blum (1989). Tc of *T. aestivum* plants infected with *Mycosphaerella graminicola* (causal agent of *Septoria tritici* blotch), can be positively correlated with disease occurrence as infected plants had higher Tc. The increase in Tc can be ascribed to the damaged cuticular layer due to infection by pathogens. Furthermore, a negative correlation was observed between Tc and leaf greenness as pathogen infection progressed. Thus, measurement of plant Tc can be used to identify both infected and un-infected areas (Eyal and Blum, 1989).

The significance of Tc under concurrent drought and pathogen infection has been shown in some studies (Pinter et al., 1979; Dow et al., 1988). When *B. vulgaris* (sugar beet) was infected with *Pythium aphanidermatum* (causal agent of root rot) under drought stress, increased Tc was observed in drought stressed plants as compared to control plants (Pinter et al., 1979). Increased Tc can be attributed to infection induced root damage, resulting in interruption of water uptake and a reduction in plant water potential. Infected plants had higher Tc as compared to the drought stressed, -uninfected plants. Similar increase in Tc was observed in *Gossypium* spp. infected with *Phymatotrichum omnivorum* (causal agent of *Phymatotrichum* root rot) under drought stress (Pinter et al., 1979). Under concurrent drought and *M. phaseolina* (causal agent of charcoal rot) infection, increased leaf temperature and decreased stomatal resistance were observed in stressed *P. vulgaris* (Mayek-Perez et al., 2002). Likewise, infection of *Sclerotinia minor* Jagger (causal agent of watery mold and soft rots) on thinned and un-thinned *Arachis hypogaea* (peanut) plants under drought stress has been studied by Dow et al. (1988). Drought stressed un-thinned plant canopy showed increased disease severity compared to the thinned treatments. Thinning lead to a modification in plant canopy size, which affected the microclimate as well as Tc. High relative humidity and microclimate associated with the un-thinned canopy favored disease infection, whereas thinned canopy exhibited lower relative humidity and lesser disease infection. The effects were further supported by a reduction in soil moisture content in the thinned fields, compared to the non-thinned ones; thinned canopy might have reduced canopy level humidity and increased transpiration rate as well as water uptake from soil, resulting in lower Tc and consequently lesser infections. Thus increasing space between the plants can be utilized as an agronomic practice in areas with less soil moisture availability for providing resistance against combined drought and pathogen infection. It has been demonstrated that plants which regulate transpiration and gas exchange could maintain cooler canopies under drought conditions. Tc has also been found to increase with increasing number of dead leaves (Blum, 2009), further emphasizing the importance of transpiration and gas exchange in reducing Tc.

Overall, a combination of drought stress and pathogen infection shows a negative correlation with Tc. Plants which maintained normal Tc under these conditions did not compromise growth and yield. Thus, Tc, which is shown to be affected by both individual and combined drought and pathogen infection, can be considered as an important trait for assessing combined drought and pathogen tolerance of plants. A simple measurement of Tc using infra-red thermometers can be implemented as an efficient means to screen genotypes better adapted to grow under combined drought and pathogen infection. Some QTLs for Tc has been found to be related to root development. For example, QTL for Tc at chromosome 2B of *T. aestivum* is also the main QTL responsible for root developmental in wheat (Pinto and Reynolds, 2015). An attempt to find the relation between Tc and Cephalosporium stripe disease (CSD) of winter wheat caused by *Cephalosporium gramineum* has been recently made by Froese et al. (2016). Although the authors could not find any significant relation between QTLs for Tc and disease severity, they suggest that a better evaluation of Tc might be helpful in proving the correlation between Tc and CSD resistance. As Tc is a good indicator of water status of plants investigation to find correlation between Tc and vascular diseases can also reveal mechanisms and QTLs for resistance to combined drought and pathogen infection.

## Development of Crops with Improved Performance Under Combined Drought and Pathogen Stress

### Role of Simulation Studies in Assessing the Impact of Drought–pathogen Combination

Crop yield is determined as a net result of complex interactions among abiotic and biotic conditions, soil features and crop management practices. Several crop modeling approaches which can predict the effect of various weather conditions on crop yield can be used to devise strategies for farm planning and regional policy development. Similarly, a number of plant disease prediction models have also been developed and evaluated. For example, Garcia et al. (2008) developed and applied a geographical information system (GIS) based agro-meteorological disease model to determine the sowing dates with low climatic risk for the infection of potato late blight disease in the Andes region of Venezuela (Garcia et al., 2008).

Considering the role of biotic stress factors in determining the yield of plants, it becomes utmost important that the effect of biotic constraints are considered in addition to the abiotic factors in order to generate a more comprehensive and relevant projection of future global plant productivity under a changing climate. In order to assess the impact of combined biotic and abiotic stresses on plants, linked “climate-crop disease” models need to be developed. Few simulation studies have been attempted to link disease forecasting models to regional climatic scenarios (Oldenburg et al., 2009; Caffarraa et al., 2012). Simulation studies like these should be extended to more crops in order to assess the yield loss potential of diseases in the current scenario of climate change. This would demand intensive collaboration between climatologists, agronomists, and plant pathologists involved in disease epidemic modeling. Efforts in this direction would help in planning better strategies for improving crop productivity.

### Role of Genomic Tools for Developing Combined Drought and Pathogen Stress Tolerant Crops

A few important molecular studies have recently been employed to elucidate the molecular responses of plants against combined drought and pathogen stresses (Supplementary Table S3). These studies have not only shed light on a plant’s defense mechanism against combined stresses but also revealed some potential candidates for improvement of plant tolerance to combined stresses. Some of the important candidate genes identified so far are methionine homeostasis gene; methionine gamma lyase (AtMGL), rapid alkalinization factor-like 8 (AtRALFL8) involved in cell wall remodeling and azelaic acid induced 1 (AZI1) functioning in systemic plant immunity (Atkinson et al., 2013). Tolerance to combined drought and pathogen stress is also contributed by genes involved in crosstalk between the drought and pathogen infection associated signaling pathways. The roles of proline and polyamine metabolism in combined drought and pathogen stress tolerance of *A. thaliana* and *V. vinifera* have also been indicated by some studies (Hatmi et al., 2015; Gupta et al., 2016). The identified candidate genes can be suitably modulated to confer enhanced tolerance against the combined stresses. The modification can be done by genome editing using tools like CRISPR/Cas9 system. CRISPR/Cas9 system can also be used to modulate the transcription of the genes of interest by guiding catalytically inactive dead Cas9 (dCas9) or dCas9 fused with transcriptional repressors/activators to the promoter of a gene. Further research in this direction using the different functional genomic approaches can, thus, help in uncovering responses of plants to combined drought and pathogen stresses.

## Conclusion and Future Perspectives

**FIGURE 3:**
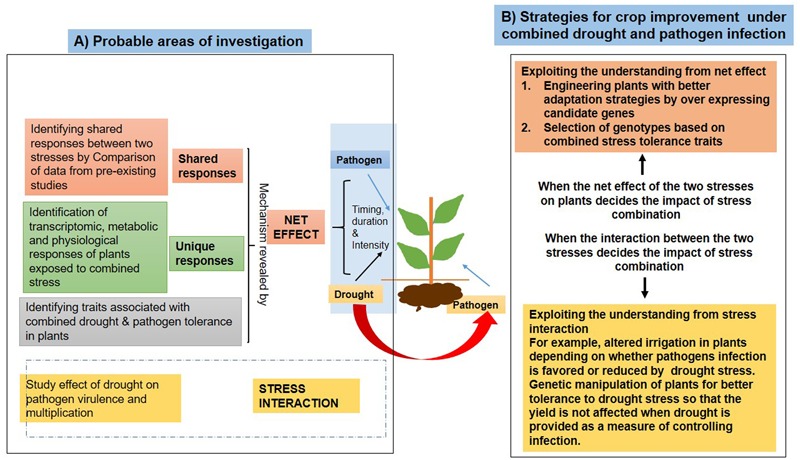
**Outline of strategies for improving crop performance under combined drought and pathogen stress.** For understanding the effect of stress on plants, it is important to first understand the nature of the stress combinations, i.e., the interaction between the two stresses as influenced by the timing, intensity, and duration. For example, the pathogen–drought stress interaction can be understood by studying the effect of drought on pathogen life-cycle and virulence. The net effect can be deciphered by studying the response of plants to combined stress which comprises of shared and unique responses. For example, a comparison of pre-existing information on a plant’s molecular responses to individual stresses (microarray datasets and metabolic profile) can help in the identification of probable shared responses. Unique responses can be studied by performing actual combined stress studies and investigating physiological, molecular, and metabolic changes in plants under the stress combinations. The other area of research can be the identification of traits associated with combined stress tolerance **(A)**. Few strategies are available for improving plant tolerance to combined stress conditions. A comprehensive understanding of the nature and effect of stress combination on plants is helpful in devising effective strategies for crop improvement under combined stress conditions. If the stress interaction is important in defining the disease incidence, strategies exploiting the stress interaction can be more helpful in enhancing tolerance of plants to combined stress. For example, a simple modulation in irrigation regime can help in combating the pathogen infection. If the net effect of both the stresses on plants is more important, the information derived from the transcriptomic studies can be utilized to select candidate genes and plants with better adaptation to combined stress can be engineered by suitable modulation of expression of the candidate genes **(B)**.

Plants under field conditions face a combination of different abiotic and biotic stresses. The interaction between these stresses and their impact on plants has been discussed earlier as part of the “disease triangle.” The interaction between the two stress conditions may either negatively or positively affect plant growth. For example, a co-existing drought can also modulate the interaction of different pathogens and plants differently, leading to either suppression or increase in pathogen growth. Therefore, it becomes very important to study the interaction between the two stresses in order to better understand the net impact of stress combinations on plants. Several important diseases such as DRR, powdery mildew, and charcoal rot are significantly affected by co-occurring drought conditions and identification and development of superior cultivars can be done if a mechanistic understanding of the interaction between pathogen and drought stress is attained. The strategies for improving crop performance under combined drought and pathogen stress have been schematically represented in **Figure 3**. Attempts to understand the interactions have already been started in the form of transcriptomic studies (Supplementary Table S3). Well-designed experiments involving simultaneous drought and pathogen stress on plants have also been undertaken, revealing some aspects of drought–pathogen interactions (Gupta et al., 2016; Sinha et al., 2016). Plant genotypes can be screened for traits such as RSA, leaf water potential, leaf pubescence, and leaf cuticular waxes for identification of superior germplasm lines.

To vividly assess the effect of different stress combinations on plants, it is imperative to design experiments that can reveal different aspects of interactions between the two stresses. A well thought about stress imposition protocol that is not very different from stresses occurring under field conditions, complemented by relevant physiological assays and the recently evolved genomic tools, can help uncover the response of plants to stress combinations. Understandings from studies on plant response to combined drought and pathogen stress can be utilized by breeders and field pathologists to better analyze the performance of the superior/tolerant genotypes. Further development of crop simulation models involving a combination of drought and pathogen stress can help in disease forecasting in places where concurrence of the two stresses is prevalent. Thus, integrative efforts from crop modeling experts, agronomists, field pathologists, breeders, physiologists, and molecular biologists can efficiently lead to development of combined stress tolerant crops that can perform well under field conditions.

## Author Contributions

MS-K conceived the concept and provided outline. PP drafted the manuscript. VI drafted “traits” part of the manuscript. MB contributed to “weeds/herbicides” part and also edited the manuscript. MS-K edited and finalized the manuscript.

## Conflict of Interest Statement

The authors declare that the research was conducted in the absence of any commercial or financial relationships that could be construed as a potential conflict of interest.
