# Supplementary material for: Impact of Combined Abiotic and Biotic Stresses on Plant Growth and Avenues for Crop Improvement by Exploiting Physio-morphological Traits
Source: Front Plant Sci. 2017 Apr 18;8:537. doi: 10.3389/fpls.2017.00537 (PMC5394115; doi:10.3389/fpls.2017.00537)
Supplement: Supplementary file 1 [file Table_1.DOC]

**Supplementary table 1: Examples of some important stress combinations affecting plant growth and yield.**

| **Sl. No.** | **Stress combination** | **Evidence of occurrence of stress interactions/geographical location** | **Crops affected** | **Impact/effect of interaction & other details** | **Reference** |
| --- | --- | --- | --- | --- | --- |
|  | **ABIOTIC-ABIOTIC STRESS INTERACTIONS** | | | | |
| 1 | Heat +Drought | Most evident combination due to global warming; experimental evidence from various crops | Many cereal crops, *Arabidopsis thaliana* | Combination negatively affected plant growth. Caused 120 billion $ in damages as compared to 20 billion $ lost due to drought stress alone. | Rizhsky et al., 2002, 2004, Mittler 2006 |
| 2 | Heat +Salinity | Evident in arid and semi-arid regions | Barley (*Hordeum vulgare*), Sorghum (*Sorghum bicolor),*  *Suaeda salsa* | Both positive and negative impact on plants were observed | Li et al., 2011 |
| 3 | Heat+ Ozone | Interaction shown by open air experiments | Silver Birch (Betula pendula) | Combined stress negatively affected tree growth | Kasurinen et al., 2012 |
| 4 | Drought +Salinity | Irrigated and dry land agricultural areas | Soybean (*Glycine max*), oilseed rape (*Brassica napus*) | Jones  and Turner 1980; Munns and Weir 1981  Both positive and negative effects were observed | Jones  and Turner 1980; Munns and Weir 1981  Munns and Weir 1981, Siddiqui et al., 2008 |
| 5 | Drought +Cold stress | Occurring in regions with continental climate like North China | Grapes (*Vitis vinifera*) | Combined stress negatively affected plant growth | Su et al., 2015 |
| 6 | Drought +Ozone | Laboratory evidence | Alfalfa (*Medicago sativa*) | Ozone ameliorated effect of drought stress | Puckette et al., 2007, Pääkkönen et al. 1998, Löw et al. 2006, Iyer et al. 2013 |
| 7 | Drought +High Light | Laboratory evidence | Pearl millet (*Pennisetum glaucum*), Sorghum | Combined stress negatively affected plant growth | Masojídek et al., 1991 |
| 8 | Salt +Ozone | Laboratory evidence | Chickpea (*Cicer arietinum*), Rice (*Oryza sativa*) | Combined stress negatively affected plant growth | Welfare et al., 1996, 2002 |
| 9 | Cold +High light | Laboratory evidence | Algae (*Dunaliella salina*) | Combined cold and high light stress cause extensive photo-oxidative stress | Haghjou et al., 2009 |
| 10 | Cold+ Ozone | Laboratory evidence | Wheat (*Triticum aestivum*) | Ozone reduced frost hardiness | Barnes and Davison, 1988, Eamus and Murray (1991) |
| 11 | Drought+ High CO_2_ | Laboratory evidence | Wheat, Kentucky grass (*Poa pratensis*) | High CO_2_ mitigated the effect of drought | Kaddour and Fuller, 2004; Song et al., 2014 |
| 12 | Salinity +High CO_2_ | Laboratory evidence | Tomato (*Solanum lycopersicum*), Broccoli  *(Brassica oleracea)* | High CO_2_ improved salt tolerance of plants | Yi et al., 2015; Zaghdoud et al., 2013 |
| 13 | Cold +High light | Evident in Mediterranean evergreens; experimental evidence for some plants available | Jojoba (*Simmondsia chinensis*) | Combined stress caused strong photo-inhibition | Loreto and Bongi 1989; Haghjau et al., 2009 |
| 14 | High light +High CO_2_ | Laboratory evidence | Lettuce (*Lactuca sativa*) | Combined stress enhanced antioxidant system of plant | Perez-Lopez et al. 2013 |
|  | **ABIOTIC-BIOTIC INTERACTIONS** | | | | |
| 1 | Drought +Pathogen | oak decline in France during drought year 1976  United States  Mediterranean regions | Trees like Oak (*Quercus robur*)  Pine (*Pinus* spp)  *Quercus* spp | Drought predisposes to oak trees to Armillaria root rot (caused by *Armillaria mellea,*  Diplodia tip blight caused by *Saphaeropsis sapinea*,  Charcoal root rot caused by *Biscogniauxia mediterranea* in oaks | Desprez-Loustau et al., 2006  Bachi and Peterson, 1985  Desprez-Loustau et al., 2006 |
|  |  |  | Potato | Drought and cyst nematode (Globodera pallid) had additive effect on plant growth |  |
| 2 | Heat +Pathogen | Prediction based | Oil seed rape (*Brassica napus*) | *Alternaria brassicae*, *Sclerotinia sclerotiorum*, and *Verticillium longisporum* are predicted to be favored by average warmer temperatures | Siebold and von Tiedemann, 2012 |
|  |  | Global circulation model (prediction based); United Kingdom | Wheat | Fusarium ear blight (caused by *Fusarium* sp.) incidence during anthesis is projected to be more severe, especially in southern England, by the 2050s (because of projected increase of temperature) | Madgwick et al. 2011 |
|  |  | Global circulation model (prediction based); Germany | Sugar beet (*Beta vulgaris*) | Early occurrence of Cercospora leaf spot (caused by *Cercospora beticola*) in sugar beet due to warmer temperatures | Richerzhagen  et al. 2011 |
|  |  | Experimental evidence, likely to occur in tropical and sub tropical countries | Tomato | *Tomato leaf spotted wilt virus* infection decreased in heat stress plants | Ghandi, A. et al. 2016 |
| 3 | Salinity+ Pathogen | Experimental evidence | Potato (*Solanum tuberosum*), Tomato, Citrus | Salinity aggravated disease caused by *Fusarium* sp, *Verticilium* and *Phytophthora* | Dzengeleski et al., 2003, Besri, 1993 |
| 4 | Cold +Pathogen | Greater losses in 1968 which had cooler summers in Arizona | Sorghum | Low temperature enhanced disease caused by *Maize dwarf mosaic virus* | Hine et al., 1970 |
| 5 | Ozone +Pathogen | Laboratory evidence | Tobacco (*Nicotiana tabaccum*) Wheat, Barley, Fescue (*Festuca arundinacea*) | A virus-resistant tobacco cultivar (*Nicotiana tabacum* L. cv. Vam) showed increased sensitivity to *Potato virus Y* at elevated O3 concentrations | Bonello et al., 1993 |
|  |  |  |  | Ozone enhanced resistance to rust in wheat, *B. sorokiniana* in barley and fescue, *Pseudomonas glycinea* in soybean, *Erysiphe polygoni* in pea | Tiedemann and Firsching , 2000, Laurence and Wood, 1978; Rusch and Laurence,1993; Plazek 2001 |
|  |  |  |  | O3 treatment conferred susceptibility to *Heterobasidion annosum* in pine seedlings | Pollastrini, et al, 2015 |
|  | **BIOTIC- BIOTIC INTERACTIONS** | | | | |
| 1 | Virus-virus | Potato growing areas | Potato | *Potato virus X* (PVX) infected potato plants in combination with *Potato virus A* causing ‘crinkle’ disease;  PVX+PVS caused mosaic and rugosity; Combination of PVX and *Tobacco mosaic virus* (TMV), which causes a severe ‘streak’ disease;  Enhanced disease symptoms in comparison to individual infections in case of in case of Potato virus disease caused by mixed infection with *Sweet potato feathery mottle virus* and *Sweet potato chlorotic stunt virus* | Stevenson et al., 2001;Nyalugwe et al., 2012; Tairo et al., 2005 |
| 2 | Bacteria- bacteria | Evident in Russia | Tomato | Combination of eight bacterial species namely *Pseudomonas cichorii*, *P. corrugate*, *P. viridiflava*, *P. mediterranea*, *P. fluorescens*, *Pectobacterium atrosepticum*, *Pectobacterium carotovorum* and *Dickeya chrysanthemi* can cause tomato pith necrosis  Soft rot of broccoli is caused by combination of *Pectobacterium carotovorum*, *P. marginalis*, *P. fluorescens* and *P. viridiflava* | Canaday et al., 1991 |
| 3 | Fungus-Fungus | Riverina region of Australia | Grapevines | Co-infection of fungal species belonging to *Botryosphaeriaceae* sp. and *Ilyonectria* sp. caused severe decline of young grafted grapevines in the field | Whitelaw-Weckert et al., 2013 |
|  |  | United Kingdom | Oilseed rape | Septoria leaf blotches caused by combination of *Septoria tritici* and *Stagonospora nodorum*;  Phoma stem canker on oilseed rape caused by combination of *Leptosphaeria biglobosa* and *L. maculans*. | Fitt et al., 2006 |
|  |  | Poland, United States, Brazil | Wheat | Foot and crown rot of wheat caused by combination of *Fusarium graminearum*, *F. culmorum*, *F. poae*, and *F. sporotrichioides* | Kuzdraliński et al., 2014; |
|  |  |  | Wheat | Fusarium head blight caused by combination of 16 species of *Fusarium* | McMullen et al., 2012; Del Ponte et al., 2014 |
|  |  | Laboratory evidence | Pea (*Pisum sativum*) | Black spot disease of pea caused by combination of 7 fungal pathogens including *Mycosphaerella pinodes*, *Phoma medicaginis*, *Phoma herbarum*, *Boerema exigua* var. exigua, and *Phoma glomerata* | Le May et al., 2009;  Lamichhane and Venturi 2015; |
|  |  | Himachal Pradesh, India | Pea | Previous inoculation with *F. oxysporum* decreases the necrotic symptoms caused by *Phoma medicaginis* var. pinodella in pea | Sagar and Sugha 1997 |
| 4 | Fungus Bacteria | Laboratory evidence | Walnut (*Juglans regia*) | Brown apical necrosis of walnut fruit caused by combination of fungi namely *Fusarium*, *Alternaria*, *Cladosporium*, *Colletotrichum*, and *Phomopsis* and a bacterium *Xanthomonas arboricola* | Belisario et al., 2002 |
|  |  | China | *Panax notoginseng* | Root rot disease complex of Panax notoginseng caused by combination of fungi (*Alternaria panax*, *Alternaria tenuis*, *Cylindrocarpon destructans*, *Cylindrocarpon didynum*, *F. solani*, *F. oxysporum*, *Phytophthora cactorum*, *Phoma herbarum*, and *Rhizoctonia solani*) and bacteria (*Pseudomonas* sp. and *Ralstonia* sp.) | Miao et al., 2006; Ma et al., 2013 |
| 5 | Herbivore- Pathogen^2^ |  | Bitter dock *(Rumex obtusifolius)* | Beetle (*Gastrophysa viridula*) grazing reduces infection of *Rumex obtusifolius* by *Uromyces rumicis* | Hatcher and  Paul, 2000 |
|  |  |  | White clover (*Trifolium repens*) | White clover mosaic virus infection decreased the attractiveness of white clover plants for herbivore (Bradysia sp) | Molken et al., 2012 |
|  |  |  | *Barbarea vulgaris* | Plants exposed to combined infection with oomycete Albugo sp. and a flea beetle, *Phyllotreta nemorum* showed higher concentrations of fungal DNA in plants exposed to herbivory; similarly, flea beetle larvae caused more damage on Albugo-infected plants. | Mölken et al., 2014 |
|  |  |  | Geranium *(Pelargonium x hortorum)* | Fungus gnat (*Bradysia impatiens*) feeding inhibit *Pythium aphanidermatum* infection of geranium seedlings. | Braun et al., 2009 |
|  |  |  | *Mimulus guttatus* | Combined infection with spittlebugs and virus showed variable effects. In some families of host plant, herbivory and infection strongly reduced plant fitness, while in others herbivory and infection influenced plants fitness to a much less extent. | Eubanks et al., 2005 |
|  |  |  | *Rumex*  *crispus* and *R. obtusifolius* seedlings | The combined effects of beetle (*Gastrophysa viridula*) and rust infection by *Uromyces rumicis* were additive, leading to 84-88% reduction in shoot dry weight. | Hatcher, 1996 |
|  |  |  | *Medicago saliva* (Laboratory evidence) | A combination of clover root curculio, *Sitona hispidula*, *Fusarium oxysporum* significantly reduced the growth of alfalfa plants. A higher level of Fusarium wilt was observed *when S. hispidula* was present than when it was absent. *C. insidiosum* did not reduce growth of alfalfa. No relation between effects of *S. hispidula* and *C. insidiosum* was detected. | Hill et al., 1969 |
|  |  | New York (1983) | Maize (*Zea mays*) | Even minimal stalk damage by *Ostrinia nubilalis* significantly predisposed plants to anthracnose stalk rot caused by *Colletotrichum graminicola*. | Keller et al., 1986 |
|  |  | Green house experiment  Green house experiment | *Medicago sativa*  *Medicago sativa* | Significantly more plants died after subjection to the combined stress of feeding by potato leafhoppers (*Empoasca fabae*), root inoculations with *F. roseum*, and exposure to winter  *Spissistilus festinus* nymphs also were found to cause a significant increase in Fusarium crown-rot (caused by *Fusarium oxysporum*) severity after six harvest periods. | Leath, and Byers, 1977  Moellenbeck et al., 1992 |
|  |  |  | *Vicia faba* | Concomitant infection with black bean aphid (*Aphis fabae*) and broad bean rust (*Uromyces viciae-fabae*) resulted in additive damage. | Pruter and Zebitz, 1991 |
|  |  | Laboratory evidence | *Glycine max* | Lengths of canker (caused by *Diaporthe phaseolorum*) on soybean plants with stem girdles induced by hopper *Spissistilus festinus* were significantly greater than in case of non-girdled plants | Russin et al., 1986 |

^2^For further information, readers are advised to refer to the extensive reviews by Lamichhane and Venturi 2015

**References**

Bachi, P.R. and Peterson J. L. (1985) Enhancement of *Sphaeropsis sapinea* stem invasion of pines by water deficits, *Plant Dis*. 69, 798–799.

Barnes, J.D., and Davison, A.W. (1988). The influence of ozone on the winter hardiness of Norway Spruce [*Picea abies* (L) Karst]. *New Phytol.* 108 (2),159-166.

Belisario, A., Maccaroni, M., Coramusi, A., Corazza, L., Pryor, B. M. and Figuli, P. (2004). First report of Alternaria species groups involved in disease complexes of hazelnut and walnut fruit. *Plant Dis.* 88 426–426.

Bonello, P., Heller, W., Sandermann, H. (1993). Ozone effects on root disease, susceptibility and defence responses in mycorrhizal and non-mycorrhizal seedling of Scots pine (*Pinus sylvestris* L.). *New Phytol.* 124, 653–663.

Braun, S.E., Sanderson, J.P., Nelson, E.B., Daughtrey, M.L. and Wraight, S.P. (2009) Fungus Gnat Feeding and Mechanical wounding inhibit *Pythium aphanidermatum* infection of geranium seedlings. *Phytopathology* 99, 1421-1428.

Canaday, C. H., Wyatt, J. E. and Mullins, J. A. (1991). Resistance of broccoli to bacterial soft rot caused by *Pseudomonas marginalis* and fluorescent Pseudomonas species. *Plant Dis.* 75, 715–720.

Del Ponte, E. M., Spolti, P., Ward, T. J., Gomes, L. B., Nicolli, C. P., Kuhnem, P. R., Silva, C.N. and Tessmann, D.J. (2014). Regional and field-specific factors affect the composition of Fusarium head blight pathogens in subtropical no-till wheat agroecosystem of Brazil. *Phytopathology* 105, 246–254.

Desprez-Loustau, M. L., Marcais, B., Nageleisen, L. M., Piou, D., and Vannini, A. (2006). Interactive effects of drought and pathogens in forest trees. *Ann Forest Sci.,* 63(6), 597-612.

Dzengeleski, S., Da Rocha, A. B.; Kirk, W.W. and Hammerschmidt, R. (2003) Effect of soil salinity and *Fusarium sambucinum* infection on development of potatoes cultivar 'Atlantic'. *Acta Hort.* 619, 251-261.

Besri, M. and Afailal, A. (1993). Effect of soil and water salt content on the development of Verticillium wilt on resistant tomato cultivars. Proc. of the 6th Intl Congress on Plant Pathology, Jul. 28-Aug. 6, Montreal, Canada.

Pekhtereva, E.S., Kornev, K.P., Matveeva, E.V., Polityko, V.A., Budenkov, N.I., Ignatov, A.N., Schaad-Pekhtereva N.W.(2009) Proc. II Intl. Symposium on Tomato Diseases, eds.: F.L. Saygili. Acta Hort. 808.

Eamus, D. and Murray, M. (1991) Photosynthetic and stomatal conductance responses of Norway spruce and beech to ozone, acid mist and frost--a conceptual model. *Environ Pollut.* 72(1), 23-44.

Eubanks, M.D., Carr, D.E. and Murphy, J.F. (2005) Variation in the response of Mimulus guttatus (Scrophulariaceae) to herbivore and virus attack. *Evol. Ecol*., 19, 15-27.

Fitt, B.D., Huang, Y.J., van den Bosch, F. and West, J.S. (2006) Coexistence of related pathogen species on arable crops in space and time. *Annu Rev Phytopathol.,* 44, 163-82.

Ghandi, A., Adi, M., Lilia, F., Linoy, A., Or, R., Mikhail, K., Mouhammad, Z., Henryk, C. and Rena, G. (2016) Tomato yellow leaf curl virus infection mitigates the heat stress response of plants grown at high temperatures. *Sci. Rep.* 6, 19715.

Haghjou, M.M., Shariati, M. and Smirnoff, N. (2009).The effect of acute high light and low temperature stresses on the ascorbate–glutathione cycle and superoxide dismutase activity in two *Dunaliella salina* strains. *Physiol. Plant.* 135, 272–280.

Hatcher, P.E. and Paul, N.D. (2000) Beetle grazing reduces natural infection of *Rumex obtusifolius* by fungal pathogens. *New Phytol.* 146, 325–333.

Hatcher, P.E. (1996). The effect of insect-fungus interactions on the autumn growth and over-wintering of Rumex crispus and *R. obtusifolius* seedlings. *J. Ecol.,* 84, 101-109.

Hill, R.R., Newton, R.C., Zeiders, K.E. and Elgin, J.H. (1969) Relationships of clover root curculio, fusarium wilt, and bacterial wilt in alfalfa. *Crop Sci.,* 9, 327-329.

Hine, R. B., Osborne, W. E. and Dennis, R. E. (1970). Elevation and temperature effects on severity of maize dwarf mosaic virus in sorghum in Arizona. *Plant Dis. Rep.* 54, 1064-1068.

Iyer, N.J., Tang, Y. and Mahalingam, R. (2013) Physiological, biochemical and molecular responses to a combination of drought and ozone in *Medicago truncatula. Plant Cell Environ*. 36(3), 706-20.

Kaddour, A. A., and Fuller, M. P. (2004). The effect of elevated CO2 and drought on the vegetative growth and development of Durum wheat (*Triticum durum* Desf.) cultivars. *Cereal Res. Comm*. 32(2), 225-232.

Kasurinen, A., Biasi, C., Holopainen, T., Rousi, M., Maenpaa, M. and Oksanen, E. (2012) Interactive effects of elevated ozone and temperature on carbon allocation of silver birch (*Betula pendula*) genotypes in an open-air field exposure. *Tree Physiol.* 32, 737–751

Keller, N.P., Bergstrom, G.C. and Carruthers, R.I. (1986) Potential yield reductions in maize associated with an anthracnose European corn-borer pest complex in New York. *Phytopathology*, 76, 586-589.

Kuzdraliński, A., Szczerba, H., Tofil, K., Filipiak, A., Garbarczyk, E., Dziadko, P., Muszyńska, M. and Solarska, E. (2014). Early PCR-based detection of *Fusarium culmorum*, *F. graminearum*, F. *sporotrichioides* and *F. poae* on stem bases of winter wheat throughout Poland*. Eur. J. Plant Pathol.* 140, 491–502.

Lamichhane, J. R., and Venturi, V. (2015). Synergisms between microbial pathogens in plant disease complexes: a growing trend. *Front. Plant Sci.,* 6, 385.

Laurence, J. A., and Wood, F. A. (1978). Effects of ozone on infection of soybean by *Pseudomonas glycinea*. *Phytopathology* 68, 441-445.

Le May, C., Potage, G., Andrivon, D., Tivoli, B. and Outreman, Y. (2009).Plant disease complex: antagonism and synergism between pathogens of the Ascochyta blight complex on Pea. J. Phytopathol. 157, 715–721.

Leath, K.T. and Byers, R.A. (1977) Interaction of Fusarium root-rot with pea aphid and potato leafhopper feeding on forage legumes. *Phytopathology,* 67, 226-229.

Li, W., Zhang, C., Lu, Q., Wen, X. and Lu, C. (2011). The combined effect of salt stress and heat shock on proteome profiling in *Suaeda salsa*. *J.Plant Physiol.* 168, 1743–1752.

Loreto, F. and Bongi G. (1989). Combined Low Temperature-High Light Effects on Gas Exchange Properties of Jojoba Leaves. *Plant Physiol*. 91, 1580-1585.

Low, M., Herbinger, K., Nunn, A.J., Haberle, K.H., Leuchner, M., Heerdt, C., Werner, H., Wipfler, P., Pretzsch, H., Tausz, M. and Matyssek, R. (2006) Extraordinary drought of 2003 overrules ozone impact on adult beech trees (*Fagus sylvatica*). *Trees,* 20(5), 539-548.

Ma, L., Cao, Y. H., Cheng, M. H., Huang, Y., Mo, M. H., Wang, Y., Yang, J.Z. and Yang, F.X. (2013). Phylogenetic diversity of bacterial endophytes of *Panax notoginseng* with antagonistic characteristics towards pathogens of root-rot disease complex. *Antonie Van Leeuwenhoek* 113, 299–312.

Madgwick, J., West, J., White, R., Semenov, M., Townsend, J., Turner, J., and Fitt, B.L. (2011). Impacts of climate change on wheat anthesis and fusarium ear blight in the UK. *Eur. J Plant Pathol*. 130, 117–131.

Masojídek, J., Trivedi, S., Halshaw, L., Alexiou, A. and Hall, D.O. (1991).The synergistic effect of drought and light stresses in sorghum and pearl millet. *Plant Physiol.* 96(1), 198-207.

McMullen, M. P., Bergstrom, G. C., De Wolf, E., Dill-Macky, R., Hershman, D., Shaner, G. and Sanford, D.V. (2012). A unified effort to fight an enemy of wheat and barley: Fusarium head blight. *Plant Dis*. 96, 1712–1728.

Miao Z. Q., Li S. D., Liu X. Z., Chen Y., Li Y., Wang Y., Guo, R.J., Xia, Z.Y. and Zhang, K.Q. (2006). The causal microorganisms of *Panax notoginseng* root rot disease. *Sci. Agric. Sinica* 39, 1371–1378.

Mittler, R. (2006). Abiotic stress, the ﬁeld environment and stress combination. *Trends* *Plant Sci.* 11, 15–19.

Moellenbeck, D.J., Quisenberry, S.S. and Colyer, P.D. (1992) Fusarium crown-rot development in alfalfa stressed by three cornered alfalfa hopper (Homoptera, Membracidae) feeding. *J Econ Entomol*., 85, 1442-1449.

Van Molken, T., de Caluwe, H., Hordijk, C. A., Leon-Reyes, A., Snoeren, T. A. L., van Dam, N. M., and Stuefer, J. F. (2012). Virus infection decreases the attractiveness of white clover plants for a non-vectoring herbivore.*Oecologia*, 170(2), 433–444.

Van Mölken, T., Kuzina, V., Munk, K. R., Olsen, C. E., Sundelin, T., van Dam, N. M. and Hauser, T. P (2014).. Consequences of combined herbivore feeding and pathogen infection for fitness of Barbarea vulgaris plants. *Oecologia*. 175(2), 589-600.

Munns, R. and Weir, R. (1981) Contribution of sugars toosmotic adjustment in elongating andexpanding zones of wheat leaves during moderate water deficits at two light levels. *Aust J Plant Physiol.* 8, 93- 105.

Nyalugwe, E.P., Wilson, C.R., Coutts, B.A. and Jones, R.A.C. (2012). Biological properties of Potato virus X in potato: effects of mixed infection with Potato virus S and resistance phenotypes in cultivars from three continents. *Plant Dis.* 96, 43–54.

Paakkonen, E., Vahala, J., Pohjola, M., Holopainen, T. and Karenlampi, L. (1998) Physiological, stomatal and ultrastructural ozone responses in birch (*Betula pendula* Roth) are modified by water stress. *Plant Cell Environ.* 21, 671–684.

Perez-Lopez, U., Miranda-Apodaca, J., Munoz-Rueda, A. and Mena-Petite, A. (2013) Lettuce production and antioxidant capacity are differentially modified by salt stress and light intensity under ambient and elevated CO_2_. *J Plant Physiol.* 170, 1517–1525.

Plazek, A., Hura, K. and Rapacz, H. (2001). The influence of ozone fumigation on metabolic efficiency and plant resistance to fungal pathogens. *J App. Bot*. 75, 8–13.

Pruter, C. and Zebitz, C.P.W. (1991) Effects of *Aphis fabae* and *Uromyces viciae-favae* on the growth of a susceptible and an aphid resistant cultivar of *Vicia faba*. *Ann. App. Biol*., 119, 215-226.

Puckette, M.C., Weng, H. and Mahalingam, R. (2007) Physiological and biochemical responses to acute ozone-induced oxidative stress in *Medicago truncatula*. *Plant Physiol Biochem*. 45, 70–79.

Richerzhagen, D., Racca, P., Zeuner, T., Kuhn, C., Falke, K., Kleinhenz, B., and Hau, B. (2011). Impact of climate change on the temporal and regional occurrence of Cercospora leaf spot in Lower Saxony. *J Plant Dis Protect.* 118(5), 168-177.

Rizhsky, L., Liang, H. and Mittler, R. (2002).The combined eﬀect of drought stress and heat shock on gene expression in tobacco. *Plant Physiol.* 130, 1143–1151

Rizhsky, L., Liang, H., Shuman, J., Shulaev, V., Davletova, S., and Mittler, R. (2004). When defense pathways collide. The response of Arabidopsis to a combination of drought and heat stress. *Plant Physiol.* 134, 1683–1696.

Rusch, H. and Laurence, J.A. (1993) Interactive effects of ozone and powdery mildew on pea seedlings. *Phytopathology* 83, 1258-1263.

Russin, J.S., Boethel, D.J., Berggren, G.T. and Snow, J.P. (1986) Effects of girdling by the three-cornered alfalfa hopper on symptom expression of soybean stem canker and associated soybean yields. *Plant Dis*., 70, 759-761.

Sagar, V. and Sugha, S. K. (1997). Role of individual and combined inocula on the development of pea root rot. *Indian Phytopathol*. 50, 499–503.

Siddiqui, Z. S., Khan, M. A., Kim, B. G., Huang, J. S. and Kwon, T. R. (2008). Physiological responses of Brassica napus genotypes to combined drought and salt stress. *Plant Stress*, 2(1), 78-83.

Siebold, M., and von Tiedemann, A. (2012). Potential effects of global warming on oilseed rape pathogens in Northern Germany. *Fungal Eco.,* 5, 62–72.

Song, Y., Yu, J. and Huang, B. (2014) Elevated CO2-mitigation of high temperature stress associated with maintenance of positive carbon balance and carbohydrate accumulation in Kentucky Bluegrass. *PLoS One* 9(3): e89725.

Su, L., Dai, Z., Li, S. and Xin, H. (2015) A novel system for evaluating drought–cold tolerance of grapevines using chlorophyll fluorescence. *BMC Plant Biol*. 15, 82.

Fasan, T. and Haverkor, A. J. (1991). The influence of cyst nematodes and drought on potato growth. 1. Effects on plant growth under semi-controlled conditions. *Netherlands J Plant Pathol.* 97(3) 151-161.

Tairo, F., Jones, R. A. C. and Valkonen, J. P. T. (2006). Potyvirus complexes in sweet potato: occurrence in Australia, serological and molecular resolution, and analysis of the Sweet potato virus 2 (SPV2) component*. Plant Dis*. 90, 1120–1128.

Tiedemann, A.V. and Firsching, K.H.( 2000). Interactive effects of elevated ozone and carbon dioxide on growth and yield of leaf rust-infected versus non-infected wheat. *Environ. Pollut.* 108, 357–363.

Welfare, K., Flowers, T. J., Taylor, G., and Yeo, A. R. (1996). Additive and antagonistic effects of ozone and salinity on the growth, ion contents and gas exchange of five varieties of rice (*Oryza sativa* L.). *Environ. Pollut.* 92(3), 257-266.

Welfare, K., Yeo, A. R., and Flowers, T. J. (2002). Effects of salinity and ozone, individually and in combination, on the growth and ion contents of two chickpea (*Cicer arietinum* L.) varieties. *Environ. Pollut.* 120(2), 397-403.

Whitelaw-Weckert, M. A., Rahman, L., Appleby, L. M., Hall A., Clark A. C., Waite H., and Hardie, W.J. (2013). Co-infection by Botryosphaeriaceae and *Ilyonectria* spp. fungi during propagation causes decline of young grafted grapevines. *Plant Pathol.* 62, 1226–1237.

Pollastrini, M., Luchi, N., Michelozzi, M., Gerosa, G., Marzuoli, R., Bussotti, F., and Capretti, P. (2015). Early physiological responses of *Pinus pinea* L. seedlings infected by *Heterobasidion* sp. pl. in an ozone-enriched atmospheric environment. *Tree Physiol.* 35(3), 331-340.

Yi, C., Yao, K., Cai, S., Li, H., Zhou, J., Xia, X. and Zhou, Y. (2015). High atmospheric carbon dioxide-dependent alleviation of salt stress is linked to Respiratory burst oxidase 1 (RBOH1)-dependent H_2_O_2_ production in tomato (*Solanum lycopersicum*). *J Exp.Bot.* erv435.

Zaghdoud, C., Mota-Cadena,s C., Carvajal, M., Muries, B., Ferchichi, A., Martinez-Ballesta, M.D. (2013). Elevated CO_2_ alleviates negative effects of salinity on broccoli (*Brassica oleracea* L. var Italica) plants by modulating water balance through aquaporins abundance. *Environ Exp. Bot.* 95, 15–24.
