# Supplementary material for: Impact of Combined Abiotic and Biotic Stresses on Plant Growth and Avenues for Crop Improvement by Exploiting Physio-morphological Traits
Source: Front Plant Sci. 2017 Apr 18;8:537. doi: 10.3389/fpls.2017.00537 (PMC5394115; doi:10.3389/fpls.2017.00537)
Supplement: Supplementary file 2 [file Table_2.DOC]

**Supplementary Table S2. Different abiotic- biotic interactions and their effects on plants.**

|  | **Name of disease** | **Causal organism** | **Abiotic stress** | **Outcome of interact-tion** | **Plant** | **Details of effect of interaction on plants** | **Reference^1^** |
| --- | --- | --- | --- | --- | --- | --- | --- |
| 1. | **FUNGUS** | | | | | | |
| 1. i) | Ramularia leaf spot | Ramularia collocygni | High light | Additive | *Hordeum vulgare* | High light aggravated the infection | Makepeace et al., 2008 |
| 1. ii) | Grey mold | Botrytis cinerea | Drought | Antagon-istic | *Solanum lycopersicum* | Drought reduced infection | Achuo et al., 2006 |
|  | Rust | *Puccinia jaceae* | Drought | Additive | *Centaurea solstitialis* | Enhanced susceptibility under drought | Shishkoff and Bruckart, 1996 |
|  | Powdery mildew | *Blumeria graminis* | Drought | Antagon-istic | *H. vulgare* | Drought promoted papillae mediated resistance | Wiese et al., 2004 |
|  | Powdery Mildew | Oidium neolycopersici | Drought | Antagon-istic | *S. lycopersicum* | Drought reduced infection | Achuo et al., 2006 |
|  | Rust | Puccinia recondite | Drought | Additive | *Triticum aestivum* | More reduced plant growth under combined stress | Bethenod et al., 2001 |
|  | Rust | Puccinia lagenophorae cooke | Drought | Additive | *Senecio vulgaris* | Drought enhanced disease symptoms | Paul and Ayres 1987 |
|  | Verticillium wilt | *Verticillium-albo-atrm* | Drought | Antagon-istic | *Medicago sativa* | Drought stress enhanced resistance to pathogen | Pennypacker et al 1991 |
|  | Powdery Mildew of barley | *Ervsiphe araininis f.sp. hordei* | Drought | Additive | *H. vulgare* | Drought and powdery mildew had additive negative effect on barley causing serious losses when occurring in combination | Ayres and Zadocks, 1979 |
|  | Common smut | *Ustilago maydis* | Drought | Additive | *Zea mays* | Warm and/or drier season predicted to increases disease | Boland et al., 2004 |
|  | Wilt | *Fusarium oxysporum ciceri* | Drought | Antagonistic | *Cicer arietinum* | Decreased soil moisture decreased the wilt symptoms | Bhatti et al., 1992 |
|  | Wilt | Fusarium oxysporum f. sp. lycoperseci | Drought | Additive | *S. lycopersicum* | Early appearance of symptoms in drought stressed plants | Ghaemi et al., 2009 |
|  |  |  |  | Antagon-istic |  | Mild drought stress induced resistance in tomato plants |  |
|  | Charcoal root rot | Macrophomina phaseolina | Salinity, drought | Additive | *Sorghum bicolor* | Drought and salinity stress predisposed plants to disease | Edmunds 1965, Waller 1986, Meyek-Perez et al., 2002; Goudarzi et al., 2011 |
|  | Wilt | Fusarium oxysporum f.sp. vasinfectum | Drought | Additive | *Gossypium hirsutum* | Drought reduced resistance disease | Ragazzi et al., 1995 |
|  |  |  | Salinity |  |  | Salinity increased severity of disease | Turco et al., 2002 |
|  | Powdery Mildew | Botrytis cinerea | Salinity | Additive | *Solanum habrochaites* | Salinity increased susceptibility of plants to the pathogen | Kissoudis et al., 2015, 2016 |
|  | Wilt | Fusarium oxysporum | Salinity | Additive | *S. lycopersicum* | Salinity enhanced severity of wilt disease | Daami-Remadi et al., 2009 |
|  | Vericillium wilt | Verticillium dahliaeis | Salinity | Additive | *S. lycopersicum* | Salinity aggravated the disease | Besri and Afailal, 1993 |
|  | Dry rot of potatoes | *Fusarium sambucinum* | Salinity | Additive | *Solanum tuberosum* | Salinity aggravated the disease | Dzengeleski et al., 2003 |
|  | Fusarium wilt of tomato | Fusarium oxysporum | Salinity | Additive | *S. lycopersicum* | Salinity aggravated the disease | Besri, 1993, Daami-Remadi, 2009 |
|  | Root rot | *Pythium aphanidermatum* | Salinity | Additive | *Agrostis spp* | Salinity predisposed plants to root rot. | Rasmussen and Stanghellini, 1988 |
|  | Rice Blast | Magnaporthe oryzae | CO_2_ | Additive | *Oryza sativa* | High CO_2_ increased susceptibility to disease | Gória et al., 2013 |
|  | Stewarts Disease | *Erwinia stewartii* | Heat, drought | Additive | *Zea mays* | Warm and/or drier season predicted to increases disease due to increase in vector population | Boland et al., 2004 |
|  | Powdery Mildew | *Podosphaera leucotricha* | Heat, drought | Additive | *Malus domestica* | Warm and/or drier season predicted to increase disease | Boland et al., 2004 |
|  | Powdery Mildew | *Uncinula necator* | Heat, drought | Additive | *Vitis vinifera* | Warm and/or drier season predicted to increase disease | Boland et al., 2004 |
|  | Powdery Mildew | *Sphaerotheca macularis* | Heat, drought | Additive | *Fragaria × ananassa* | Warm and/or drier season predicted to increase disease | Boland et al., 2004 |
|  | Dry root rot | *Rhizoctonia bataticola* | Heat, Drought | Additive | *Cicer arietinum* | A combination of high temperature and drought predisposed chickpea to dry rot rot | Sharma et al., 2013 |
| 2 | ***OOMYCETES*** | | | | | | |
|  | Brown fruit rot | Phytophthora parasitica and P. citrophthorais | Salinity | Additive | *Citrus sp, S. lycopersicum* | Salinity aggravated the disease | Besri, 1993, Snapp et al., 1991 |
|  | Root rot | Phytophthora cryptogea | Salinity | Additive | *Chrysanthemum spp* | Salinity aggravated the disease | Besri and Afailal, 1993 |
| 3 | ***BACTERIA*** | | | | | | |
|  | Bacterial Speck | *Pseudomonas syringae* | Drought | Antagon-istic | *Arabidopsis thaliana, S. lycopersicum* | Drought stress provided endurance to the bacterial pathogen; reduced disease development due to less rain | Gupta et al 2016, Boland et al., 2004 |
|  | Fire blight of apple | *Erwinia amylovora* | Drought | Antagonistic | *Malus domestica* | Drought reduced disease development | Sholberg and Boulé, 2009 |
|  | Wilt | *Xanthomonas campestris* pv. *musacearum* | Drought | Additive | *Musa paradisiaca* | Drought stress made the plants susceptible to disease | Ochola et al., 2014 |
|  | Bacterial leaf scorch | *Xylella fastidiosa* | Drought | Additive | *Partheno-cissus quinquefolia* | Drought stress made the plants susceptible to disease | McElrone et al., 2001, Choi et al., 2013 |
|  | Ralstonia wilt | *Ralstonia solanacearum* | Temperature | Additive | *S. lycopersicum* | High soil temperature increased infection by the pathogen | Kůdela, 2009, Ahanger 2013 |
| 4. | ***VIRUS*** | | | | | | |
|  | Mottling and crinkling | Eggplant mottled crinkle virus | Salinity | Antagonistic | *Nicotiana benthamiana* | Salinity stress reduced viral accumulation in plants | Moldakimova et al., 2012 |
|  | Mosaic | *Cucumber Mosaic virus* | Drought (prediction) | Antagonistic | *Beta vulgaris, Citrullus lanatus,*  *Cucumis sativus* | Virus infection increases drought tolerance; warmer and drier summers predicted to increase the disease | Xu et al., 2008; Boland et al., 2004. |
|  | Mosaic | TMV | Heat | Additive | *A. thaliana* | Heat enhanced susceptibility to viral infection | Prasch and Sonnewald, 2013 |
|  | Tomato spotted wilt | *Tomato spotted wilt virus* | Heat | Additive | *Capsicum chinense* | Heat stress reduced resistance of plants to the virus | Moury et al., 1998 |
|  | Tomato leaf curl | *Tomato yellow leaf curl virus* | Heat | Additive | *S. lycopersicum* | Viral infection reduced heat stress tolerance of plants | Anfoka et al., 2016 |
|  | Turnip crinkle | *Turnip crinkle virus* (TCV) | Heat | Additive | *A. thaliana* | High temperature enhanced multiplication of the virus | Zhang et al., 2012 |
|  | Potato interveinal mosaic | *PVX-potyvirus* combination | Heat | Additive | *Solanum tuberosum* | Enhanced virulence under high temperatures | Aquilar et al., 2015 |
|  | Potato leaf roll | *Potato leaf roll virus* | Heat | Additive | *Solanum tuberosum* | Warmer temperatures predicted to increase the disease development due to increase in vectors | Boland et al., 2004 |
|  | Bean common mosaic | *Bean common mosaic virus* | Heat | Additive | *Phaseolus vulgaris* | Warmer temperatures predicted to increase the disease development due to increase in vectors | Boland et al., 2004 |
|  | Bean yellow mosaic | *Bean yellow mosaic virus* | Heat | Additive | *P. vulgaris* | Warmer temperatures predicted to increase the disease development due to increase in vectors | Boland et al., 2004 |
|  | Soybean mosaic | *Soybean mosaic virus* | Heat | Additive | *Nicotiana tabacum* | Warmer temperatures predicted to increase the disease development due to increase in vector population | Boland et al., 2004 |
|  | Tobacco ringspot | *Tobacco ringspot virus* | Heat | Additive | N. tabacum | Warmer temperatures predicted to increase the disease development due to increase in vector population | Boland et al., 2004 |
|  | Tomato mosaic | *Tomato mosaic virus* | Heat | Additive | *S. lycopersicum* | Warmer temperatures predicted to increase the disease development due to increase in vector population | Boland et al., 2004 |
|  | Barley yellow dwarf | *Barley yellow dwarf virus* | CO_2_ | Antagonistic | *Hordeum vulgare* | Decreased by increased CO2 | Malmstorm and Field, 1997 |
| 5 | **Nematodes** | | | | | | |
| 1. i. | Cyst Nematode | *Heterodera glycines* | Heat, drought | Additive | *Glycine max* | Warmer and drier regions predicted to increase disease | Boland et al., 2004 |
|  | Cyst Nematode | *Heterodera sacchari* | Drought | Additive | *O. sativa* | *H. sacchari* increased the  effects of drought and drought-related losses | Audebert et al., 2000 |
|  | Cyst Nematode | *Meloidogyne incognita* | Drought | Independent/Additive | *‎Gossypium hirsutum* | Combined stress had additive effects on plants | Davis et al., 2014 |
|  | Cyst Nematode | *Globodera pallida* | Drought | Additive | *S. tuberosum* | Drought led to higher cyst infection | Haverkort et al., 1991 |
| 6 | **Weeds** |  |  |  |  |  |  |
|  | Redroot pigweed | *Amaranthus retroflexus* | Drought | Additive | *Lycopersicon esculentum* | Drought led to increased redroot pigweed competition and reduced crop yield | Valerio et al. 2013 |
|  | Silverleaf nightshade | *Solanum elaeagnifolium* | Drought | Additive | *Gossypium hirsutum* | Drought led to increased nightshade competition and reduced crop yield | Green et al. 1987 |
|  | Jimsonweed | *Datura stramonium* | Drought | Antagonistic | *Gossypium hirsutum* | Drought led to decreased jimsonweed competition | Oliver et al. 1991 |
|  | Common cocklebur | *Xanthium strumarium* | Drought | Antagonistic | *Glycine max* | Drought led to reduced common cocklebur competition | Mortensen and Coble 1989 |
|  | Jointed goatgrass | *Aegilops cylindrical* | Drought | Neutral | *Triticum aestivum* | Drought had no impact on weed interference | Anderson 1993 |
|  | Ivyleaf morningglory | *Ipomoea hederacea* | High temperature | Additive | *Glycine max* | Warmer temperatures increased ivyleaf morningglory interference and reduced crop yield | Cordes and Bauman 1984 |
|  | Johnsongrass | *Sorghum halepense* | CO_2_ | Antagonistic | *Glycine max* | Elevated CO_2_ concentrations reduced the competitive interaction of johnsongrass (C_4_) on the crop (C_3_) | Patterson et al. 1984 |

^1^For further information, readers are advised to refer to the extensive review by Boland et al., 2004.

*additive: Both stresses resulting into a net negative impact on plants; antagonistic: The net impact of combined stress is less severe than any of the individual stress

**References**

Achuo, E. A., Prinsen, E. and Hofte, M. (2006). Inﬂuence of drought, salt stress and abscisic acid on the resistance of tomato to *Botrytis cinerea* and *Oidium neolycopersici*. *Plant Pathol.* 55, 178–186.

Aguilar, E., Allende, L., Del Toro, F.J., Chung, B.N., Canto, T. and Tenllado, F. (2015) Effects of elevated CO₂ and temperature on pathogenicity determinants and virulence of *Potato virus X*/Potyvirus-associated synergism. *Mol Plant Microbe Interact*. 28(12):1364-73.

Ahanger, R. A., Bhat, H. A., Bhat, T. A., Ganie, S. A., Lone, A. A., Wani, I. A., et al. (2013) Impact of climate change on plant diseases. *Int. J. Modern Plant Anim. Sci*., 1, 105-115.

Anderson, R.L. (1993) Jointed goatgrass (*Aegilops cylindrica*) ecology and interference in winter wheat. *Weed Sci.* 41:388-393.

Audebert, A., Coyne, D. L., Dingkuhn, M., and Plowright, R. A. (2000). The influence of cyst nematodes (*Heterodera sacchari*) and drought on water relations and growth of upland rice in Côte d'Ivoire. *Plant Soil,* 220(1-2), 235-242.

Ayres, P.G., and Zadocks, J.C. (1979). Combined effects of powdery mildew disease and soil water level on the water relations and growth of barley. *Physiol. Plant Pathol.* 14: 347-361.

Besri, M. and A. Afailal (1993). Effect of soil and water salt content on the development of Verticillium wilt on resistant tomato cultivars. Proceedings of the 6th International Congress on Plant Pathology, Jul. 28-Aug. 6, Montreal, Canada

Bethenod, O., Huber, L. and Slimi, H. (2001) Photosynthetic Response of Wheat to Stress Induced by *Puccinia recondita* and Post-Infection Drought. *Photosynthetica* 39, 581.

Bhatti, M. A., and Kraft, J. M. (1992). Influence of soil moisture on root rot and wilt of chickpea. *Plant Dis*., 76(12), 1259-1262.

Boland, G.J., Melzer, M.S. Hopkin, A. Higgins, V. and Nassuth, A. (2004) Climate change and plant diseases in Ontario. *Can. J. Plant Pathol*. 2, 335–350.

Brooks, D.H. (1972). Observations on the effects of mildew, *Ervsiohe qraminis* on the growth of spring and winter barley. *Ann. Appl. Biol*. 70, 149-156.

Choi, H.K., Iandolino, A., da Silva and F.G., Cook, D.R (2013) Water deficit modulates the response of *Vitis vinifera* to the Pierce's disease pathogen *Xylella fastidiosa*. *Mol Plant Microbe Interact*. 6, 643-57.

Cordes, R.C. and Bauman, T.T. (1984) Field competition between ivy leaf morning glory (*Ipomoea hederacea*) and soybeans (*Glycine max*). *Weed Sci.* 32, 364-370.

Daami-Remadi, M., Souissi, A., Oun, B. H., Mansour M. and Nasraoui, B. (2009) Salinity effects on Fusarium wilt severity and tomato growth. *Dyn. Soil Dyn. Plant* 3(1), 61-69.

Davis, R. F., Earl, H. J. and Timper, P (2014). Effect of simultaneous water deficit stress and *Meloidogyne incognita* infection on cotton yield and fiber quality. *J Nematol.* 46(2), 108–118.

Dzengeleski, S., Da Rocha, A. B.; Kirk, W.W. and Hammerschmidt, R. (2003) Effect of soil salinity and *Fusarium sambucinum* infection on development of potatoes cultivar 'Atlantic'. *Acta Hort.* 619, 251-261

Edmunds, L. K., Voigt, R. L. and Carasso, F. M. (1965). Charcoal rot induction and development in the field in Arizona. *Proc. Biennial Grain Sorghum Res. Util. Conf.* 4, 47-53.

Ghandi, A., Adi, M., Lilia, F., Linoy, A., Or, R., Mikhail, K., Mouhammad, Z., Henryk, C. and Rena, G. (2016*) Tomato yellow leaf curl virus* infection mitigates the heat stress response of plants grown at high temperatures. *Sci Rep*., 6, 19715.

Ghaemi, A., Rahimi, A., and Banihashemi, Z. (2009) Effects of water stress and *Fusarium oxysporum* f. sp.*lycoperseci* on growth (leaf area, plant height, shoot dry matter) and shoot nitrogen content of tomatoes under greenhouse conditions. *Iran Agric. Res.,* 28(2), 51-61.

Gória, M. M., Ghini, R., and Bettiol, W. (2013). Elevated atmospheric CO2 concentration increases rice blast severity. *Trop. Plant Pathol*., 38(3), 253-257.

Goudarzi, A., Banihashemi, Z. and Maftoun, M. (2011). Effect of salt and water stress on root infection by *Macrophomina phaseolina* and ion composition in shoot in sorghum. *Iran.* *J. Plant Pathol*. 47(3), 69-83.

Green, J.D., Murray, D.S., and Verhalen, L. M. (1987). Full season interference of silverleaf nightshade (*Solanum elaeagnifloium*) with cotton (*Gossypium hirsutum*). *Weed Sci.* 35, 813-818.

Gupta A., Dixit, S. K. and Senthil-Kumar M. (2016). Drought stress predominantly endures Arabidopsis thaliana to *Pseudomonas syringae* infection. *Front Plant Sci*. 7, 808.

Haverkort, A. J., Fasan, T., and Van de Waart, M. (1991). The influence of cyst nematodes and drought on potato growth. 2. Effects on plant water relations under semi-controlled conditions. *Net. J Plant Pathol.* 97(3), 162-170.

Kissoudis, C., Chowdhury, R., van Heusden, S., van de Wiel, C., Finkers, R., Visser, R. G., and van der Linden, G. (2015). Combined biotic and abiotic stress resistance in tomato. *Euphytica*, 202(2), 317-332.

Kissoudis, C., Sunarti, S., van de Wiel, C., Visser, R.G., van der Linden, C.G., Bai Y2 (2016) Responses to combined abiotic and biotic stress in tomato are governed by stress intensity and resistance mechanism. *J Exp Bot*.;67(17), 5119-32.

Kudela V. (2009). Potential impact of climate change on geographic distribution of plant pathogenic bacteria in central Europe. *Plant Protect.* *Sci*. 45, S27–S32.

Makepeace, J.C., Havis, N.D., Burke, J.I., Oxley, S.J.P. and Brown, J.K.M. (2008). A method of inoculating barley seedlings with *Ramularia collocygni*. *Plant Pathol.* 57, 991–999.

Malmström, C. M., and Field, C. B. (1997) Virus‐induced differences in the response of oat plants to elevated carbon dioxide. *Plant Cell Environ.* 20(2), 178-188.

Mayek-Perez, N., Garcia-Espinosa, R., Lopez-Castaneda, C., Acosta-Gallegos, J. A., and Simpson, J. (2002). Water relations, histopathology and growth of common bean (*Phaseolus vulgaris* L.) during pathogenesis of *Macrophomina phaseolina* under drought stress. *Physiol. Mol. Plant Pathol.* 60, 185–195.

McElrone, A. J., Sherald, J. L. and Forseth, I. N. (2001). Effects of water stress on symptomatology and growth of *Parthenocissus quinquefolia* infected by *Xylella fastidiosa*. *Plant Dis.* 85, 1160–4.

Moldakimova, N.A., Mukiyanova, G.S., Yarmolinsky, D.G., Brychkova, G.G. Scholthof, H.B., Sagi, M. and Omarov R.T. (2012) Effect of salinity on viral disease spread in plants. *J Stress Physiol. Biochem*. 8(3), S17

Mortensen, D.A. and Coble, H.D. (1989). The influence of soil water content on common cocklebur (*Xanthium strumarium*) interference with soybean (*Glycine max*). *Weed Sci.* 37, 76-83.

Moury, Benoît, Selassie, K. G., Marchoux, G., Daubèze, A. M., and Palloix, A. (1998) High temperature effects on hypersensitive resistance to *Tomato spotted wilt tospovirus* (TSWV) in pepper (*Capsicum chinense* Jacq.). *Eur J Plant Pathol* 104(5), 489-498.

Ochola, D., Ocimati, W., Tinzaara, W., Blomme, G., and Karamura, E. B. (2015). Effects of water stress on the development of banana Xanthomonas wilt disease. *Plant Pathol.,* 64(3), 552-558.

Oliver, L.R., Chandler J.M., and Buchanan G. A. (1991) Influence of geographic region on jimsonweed (*Datura stramonium*) interference in soybeans (*Glycine max*) and cotton (*Gossypium hirsutum*). *Weed Sci.* 39, 585-589.

Patterson, D.T., Flint, E.P. and Beyers, J.L. (1984). Effects of CO2 enrichment on competition between a C4 weed and a C3 crop. *Weed Sci.* 32,101-105.

Paul, N. D., and Ayres, P. G. (1987). Effects of rust infection of *Senecio vulgaris* on competition with lettuce. *Weed Res*., 27(6), 431-441.

Pennypacker, B.W, Leath, K.T. and Hill, Jr R.R. (1991). Impact of drought stress on the expression of resistance to *Verticillium albo*‐atrum in alfalfa. *Phytopathology* 81, 1014–1024.

Prasch, C. M., and Sonnewald, U. (2013). Simultaneous application of heat, drought, and virus to Arabidopsis plants reveals signiﬁcant shifts in signaling networks. *Plant Physiol.* 162, 1849–1866. doi: 10.1104/pp.113.221044

Ragazzi, A., Moricca, S. and Dellavalle, I. (1999). Water stress and the development of cankers by *Diplodia mutila* on *Quercus robur*. *J Phytopathol.,* 147, 425–428.

Ragazzi, A., Moricca, S., Dellavalle, I., and Mancini, F. (1995). Infection of cotton by *Fusarium oxysporum* f. sp. vasinfectum as affected by water stress. *Phytoparasitica,* 23(4), 315-321.

Rasmussen, S.L. and Stanghellini, M.E. (1988). Effect of salinity stress on development of pythium blight in *Agrostis palustris*. *Phytopathology* 78, 1495–7.

Sharma, M. and Pande, S. (2013) Unravelling effects of temperature and soil moisture stress response on development of dry root rot [*Rhizoctonia bataticola* (Taub.)] Butler in chickpea. *Am J Plant Sci*. 4, 584-589.

Shishkoff, N., and Bruckart, W. L. (1996). Water stress and damage caused by *Puccinia jaceae* on two Centaurea species. *Biol. Control*, 6(1), 57-63.

Sholberg, P. L. and Boule, J. (2009). Palmolive detergent controls apple, cherry, and grape powdery mildew. *Can J Plant Sci*., 89(6), 1139-1147.

Snapp, S. S., Shennan, C., and Bruggen, A. V. (1991). Effects of salinity on severity of infection by Phytophthora parasitica Dast. ion concentrations and growth of tomato, *Lycopersicon esculentum* Mill. *New Phytol.,* 119(2), 275-284.

Turco, E., Naldini, D. and Ragazzi, A. (2002). Disease incidence and vessel anatomy in cotton plants infected with *Fusarium oxysporum* f. sp. *vasinfectum* under salinity stress. *J Plant Dis Protect*. 109, 15-24

Valerio, M., Lovelli, S., Perniola, M., Di Tommaso, T. and Ziska, L. (2013) The role of water availability on weed–crop interactions in processing tomato for southern Italy. *Acta Agri Scand., Sec. B - Soil Plant Sci*. 63(1), 62-68.

Waller, J.M. (1986). “Drought, irrigation and fungal diseases of tropical crops” in *Water, Fungi and Plants*. eds. P.G. Ayres and L. Boddy. (Cambridge, Cambridge University Press,), 175-187.

Wiese, J., Kranz, T., and Schubert, S. (2004). Induction of pathogen resistance in barley by abiotic stress. *Plant Biol*., 6(5), 529-536

Xu, P., Chen, F., Mannas, J.P., Feldman, T., Sumner, L.W. and Roossinck, M.J. (2008) Virus infection improves drought tolerance. *New Phytol.* 180, 911–21.

Zhang, X., Zhang, X., Singh, J., Li, D. and Qu, F. (2012). Temperature-dependent survival of Turnip crinkle virus-infected arabidopsis plants relies on an RNA silencing-based defense that requires dcl2, AGO2, and HEN1. *J. Virol.* 86, 6847–6854.
