# Supplementary material for: Impact of Combined Abiotic and Biotic Stresses on Plant Growth and Avenues for Crop Improvement by Exploiting Physio-morphological Traits
Source: Front Plant Sci. 2017 Apr 18;8:537. doi: 10.3389/fpls.2017.00537 (PMC5394115; doi:10.3389/fpls.2017.00537)
Supplement: Supplementary file 3 [file Table_3.doc]

Supplementary Table 3. List of recent molecular and physiological studies on combined drought and pathogen infection.

| **Sl.No.** | **Plant** | **Stress imposed** | **Actual combined stress/Meta-analysis** | **Methodology employed** | **Major findings** | **Reference** |
| --- | --- | --- | --- | --- | --- | --- |
| 1 | *Arabidopsis thaliana* | Individual, double and triple-drought, heat and Tobacco mosaic virus infection | Actual combined stress imposition | Microarray | 11 genes were found to be unique, whereas 23 genes were found to be commonly regulated under the individual and combined stresses. | Prasch and Sonnewald, 2013 |
| 2 | *A. thaliana* | Combined drought and nematode (*Heterodera schachtii)* infection | Actual combined stress imposition | Microarray | 50 genes specifically upregulated under combined stress. Function of three genes namely *Rapid Alkalinization Factor-Like8* (*AtRALFL8*), methionine gamma lyase (AtMGL) and Azelaic acid induced1 (AZI1) studied in detail. | Atkinson et al., 2013 |
| 3 | *A. thaliana* | Combined drought and *Pseudomonas syringae* pv tomato DC3000 infection | Actual combined stress imposition | Microarray | Proline metabolism and polyamine metabolism | Gupta et al., 2016 |
| 4 | *A. thaliana* | Combined drought and *Pieris rapae* infection | Actual combined stress imposition | RNAseq | Combination of drought and *P. rapae* induced downregulation of defense associated genes | Davila Olivas et al., 2016 |
| 5 | *A. thaliana* | *In silico* analysis | No combined stress imposition | Comparison of microarray datasets on transcriptomic responses of *A. thaliana* to infection with *Botrytis cinerea* cold, drought, and oxidative stresses | 6% genes were commonly upregulated under biotic and drought stress whereas 7% genes were commonly down regulated under the two stress conditions. | Sham et al., 2014 |
| 6 | *A. thaliana* | *In silico analysis* | No combined stress imposition | RNAseq | When two stresses are applied sequentially, transcriptomic profiles of plants very similar to the second stress, irrespective of the nature of the first stress. Hormone as major players in interactions between two stresses. | Coolen et al., 2016 |
| 7 | *Oryza sativa* | Combined drought and *Xanthomonas oryzae* pv oryzae infection | Combined stress imposition | Morpho-physiological analysis of effect of combined drought and Xoo infection on rice plants | Drought stress affects the response of rice plants to Xoo in a genotype specific manner | Dossa et al., 2016 |
| 8. | *Vitis vinifera* | Combined drought and *Xyllela fastidiosa* infection | Actual combined stress imposition | Microarray | 138 transcripts differentially regulated under combined stress; upregulation of genes associated with phenylpropanoid and flavonoid biosynthesis, pathogenesis-related proteins, ABA- and JA biosynthesis pathways, and downregulation of genes involved in photosynthesis, growth, and nutrition. | Choi et al., 2013 |
| 9 | *V. vinifera* | Leaf discs from drought stressed plants exposed to *B. cinerea* infection. | Combined stress impositon | Morpho-physiological analysis of effect of combined drought and B. cinerea infection on grapevine | Drought stress induces susceptibility to *B. cinerea* infection in grapevine | Hatmi et al., 2014 |
| 10 | *Helianthus anus* | Combined drought and *Plasmopara halstedii* infection | Meta-analysis as well as actual combined stress imposition | Meta-analysis and Gene expression analysis using qRTPCR | Oxidative stress responsive genes part of shared response | Ramu et al., 2016 |

**References**

Atkinson, N.J., Lilley, C.J. and Urwin, P.E. (2013). Identification of genes involved in the response of Arabidopsis to simultaneous biotic and abiotic stresses. *Plant Physiol.*162, 2028–41.

Choi, H.-K., Iandolino, A., Goes da Silva, F. and Cook, D. (2013). Water deficit modulates the response of *Vitis* *vinifera* to the Pierce’s disease pathogen *Xylella* *fastidiosa*. *Mol Plant Microbe Interact.* 26, 643–57.

Coolen, S., Proietti, S., Hickman, R., Davila Olivas, N.H., Huang, P.P., Van Verk, M.C., Van Pelt, J.A., Wittenberg, A.H., De Vos, M., Prins, M., Van Loon, J.J., Aarts, M.G., Dicke, M., Pieterse, C.M., Van Wees, S.C. (2016). Transcriptome dynamics of Arabidopsis during sequential biotic and abiotic stresses. *Plant J.* 86(3), 249-67.

Davila Olivas, N.H., Coolen, S., Huang, P., Severing, E., van Verk, M.C., Hickman, R., Wittenberg, A.H., de Vos, M., Prins, M., van Loon, J.J., Aarts, M.G., van Wees, S.C., Pieterse, C.M. and Dicke, M. (2016). Effect of prior drought and pathogen stress on Arabidopsis transcriptome changes to caterpillar herbivory. *New Phytol.* 210(4), 1344-56.

Dossa, G.S., Torres, R., Henry, A., Oliva, R., Maiss, E., Cruz, C.V., Wydra, K. (2016). Rice response to simultaneous bacterial blight and drought stress during compatible and incompatible interactions. *Eur J Plant Pathol*. doi:10.1007/s10658-016-0985-8

Gupta, A., Sarkar, A.K. and Senthil-Kumar, M. (2016). Global transcriptional analysis reveals unique and shared responses in *Arabidopsis* *thaliana* exposed to combined drought and pathogen stress. *Front. Plant Sci*. 7, 686.

Hatmi, S., Gruau, C., Trotel-Aziz, P., Villaume, S., Rabenoelina, F., Baillieul, F., Eullaffroy, P., Clément, C., Ferchichi, A., and Aziz, A. (2015). Drought stress tolerance in grapevine involves activation of polyamine oxidation contributing to improved immune response and low susceptibility to *Botrytis cinerea*. *J Exp Bot.* 66(3), 775-87.

Prasch, C.M. and Sonnewald, U. (2013). Simultaneous application of heat, drought, and virus to Arabidopsis plants reveals significant shifts in signaling networks. Plant Physiol. 162, 1849–66.

Ramu, V.S., Paramanantham, A., Ramegowda, V., Mohan-Raju, B., Udayakumar, M. and Senthil-Kumar, M. (2016). Transcriptome analysis of sunflower genotypes with contrasting oxidative stress tolerance reveals individual and combined- biotic and abiotic stress tolerance mechanisms. *PLoS ONE* 11(6), e0157522.

Sham, A., Al-Azzawi, A., Al-Ameri, S., Al-Mahmoud, B., Awwad, F., Al-Rawashdeh, A., Iratni, R. and AbuQamar, S. (2014). Transcriptome analysis reveals genes commonly induced by *Botrytis cinerea* infection, cold, drought and oxidative stresses in Arabidopsis. PLoS One. 9(11): e113718.
