# Supplementary material for: Impact of Combined Abiotic and Biotic Stresses on Plant Growth and Avenues for Crop Improvement by Exploiting Physio-morphological Traits
Source: Front Plant Sci. 2017 Apr 18;8:537. doi: 10.3389/fpls.2017.00537 (PMC5394115; doi:10.3389/fpls.2017.00537)
Supplement: Supplementary file 4 [file Presentation_1.ppt]

## Slide 1
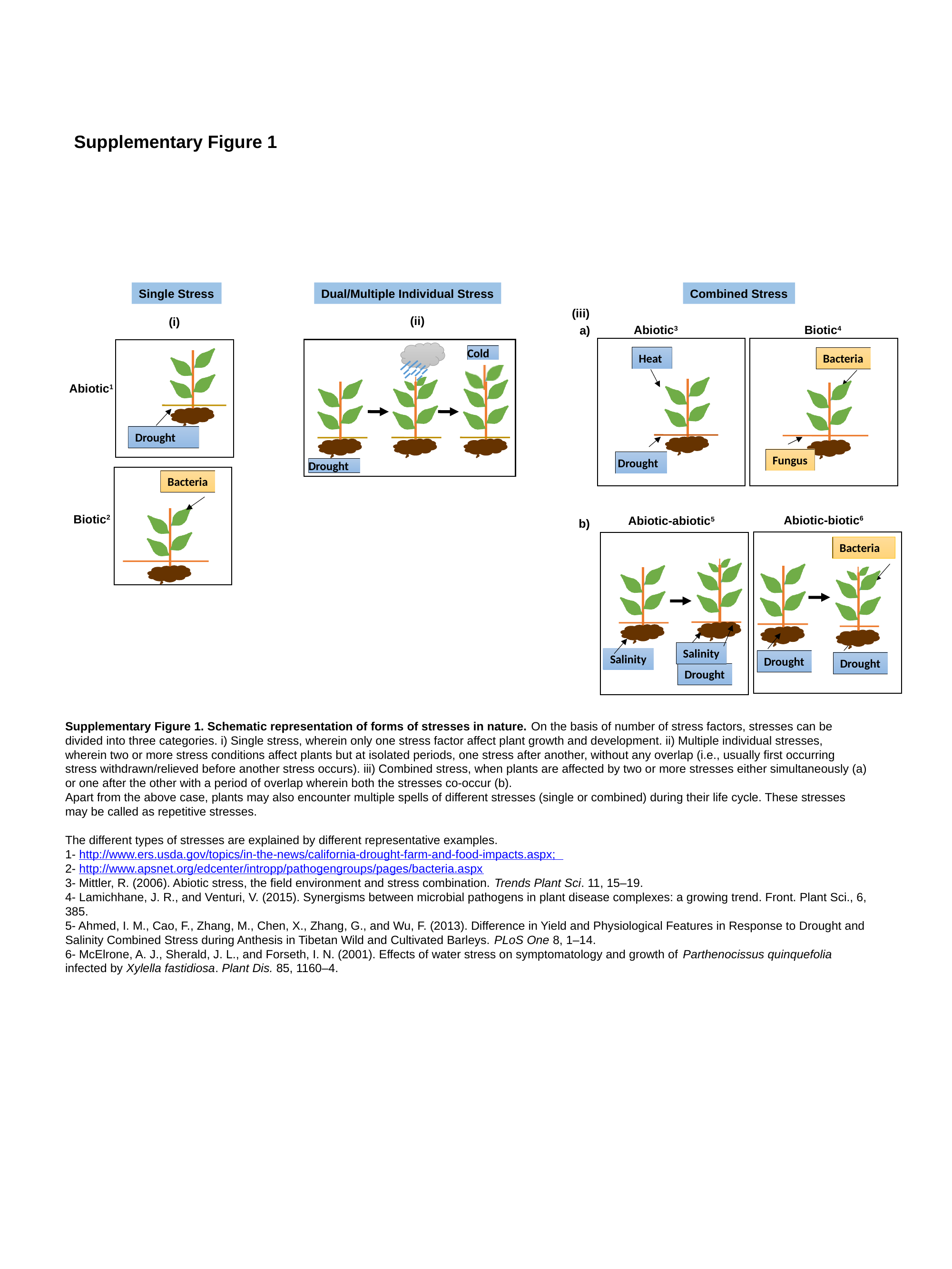

Supplementary Figure 1
Single Stress
Dual/Multiple Individual Stress
Combined Stress
 (iii)
 (ii)
 (i)
 Abiotic3
Biotic4
 a)
Bacteria
Fungus
Heat
 Drought
 Abiotic1
Drought
Cold
Drought
Bacteria
Biotic2
 Abiotic-biotic6
 Abiotic-abiotic5
 b)
Salinity
Drought
Bacteria
Salinity
Drought
Drought
Supplementary Figure 1. Schematic representation of forms of stresses in nature. On the basis of number of stress factors, stresses can be divided into three categories. i) Single stress, wherein only one stress factor affect plant growth and development. ii) Multiple individual stresses, wherein two or more stress conditions affect plants but at isolated periods, one stress after another, without any overlap (i.e., usually first occurring stress withdrawn/relieved before another stress occurs). iii) Combined stress, when plants are affected by two or more stresses either simultaneously (a) or one after the other with a period of overlap wherein both the stresses co-occur (b).
Apart from the above case, plants may also encounter multiple spells of different stresses (single or combined) during their life cycle. These stresses may be called as repetitive stresses.
The different types of stresses are explained by different representative examples.
1- http://www.ers.usda.gov/topics/in-the-news/california-drought-farm-and-food-impacts.aspx;
2- http://www.apsnet.org/edcenter/intropp/pathogengroups/pages/bacteria.aspx
3- Mittler, R. (2006). Abiotic stress, the ﬁeld environment and stress combination. Trends Plant Sci. 11, 15–19.
4- Lamichhane, J. R., and Venturi, V. (2015). Synergisms between microbial pathogens in plant disease complexes: a growing trend. Front. Plant Sci., 6, 385.
5- Ahmed, I. M., Cao, F., Zhang, M., Chen, X., Zhang, G., and Wu, F. (2013). Difference in Yield and Physiological Features in Response to Drought and Salinity Combined Stress during Anthesis in Tibetan Wild and Cultivated Barleys. PLoS One 8, 1–14.
6- McElrone, A. J., Sherald, J. L., and Forseth, I. N. (2001). Effects of water stress on symptomatology and growth of Parthenocissus quinquefolia infected by Xylella fastidiosa. Plant Dis. 85, 1160–4.

## Slide 2
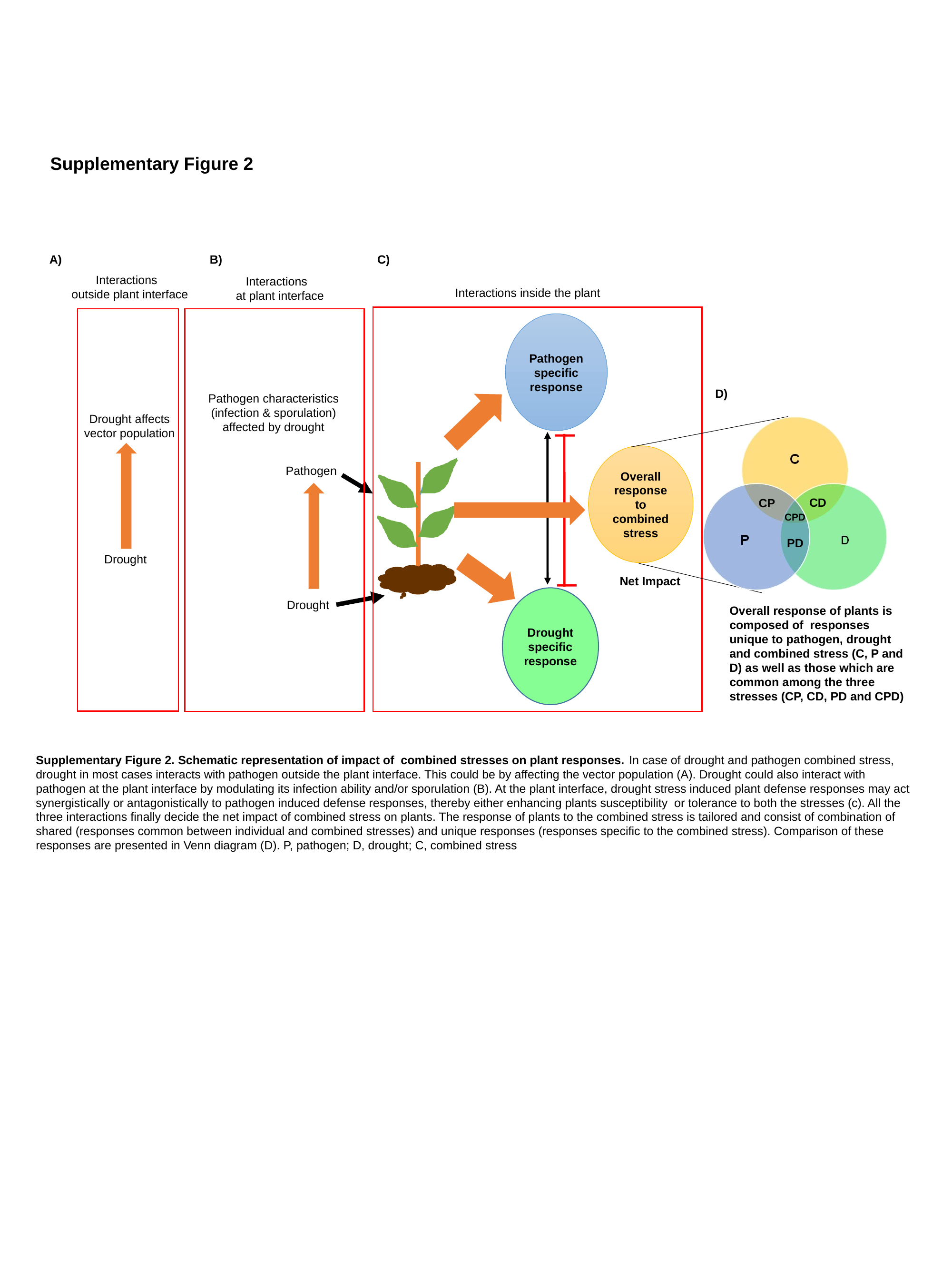

Supplementary Figure 2
A)
B)
C)
Interactions
 outside plant interface
Interactions
 at plant interface
Interactions inside the plant
Pathogen specific response
D)
Pathogen characteristics (infection & sporulation) affected by drought
Drought affects vector population
CD
CP
CPD
PD
Overall response to combined stress
Pathogen
Drought
Net Impact
Drought specific response
Drought
Overall response of plants is composed of responses unique to pathogen, drought and combined stress (C, P and D) as well as those which are common among the three stresses (CP, CD, PD and CPD)
Supplementary Figure 2. Schematic representation of impact of combined stresses on plant responses. In case of drought and pathogen combined stress, drought in most cases interacts with pathogen outside the plant interface. This could be by affecting the vector population (A). Drought could also interact with pathogen at the plant interface by modulating its infection ability and/or sporulation (B). At the plant interface, drought stress induced plant defense responses may act synergistically or antagonistically to pathogen induced defense responses, thereby either enhancing plants susceptibility or tolerance to both the stresses (c). All the three interactions finally decide the net impact of combined stress on plants. The response of plants to the combined stress is tailored and consist of combination of shared (responses common between individual and combined stresses) and unique responses (responses specific to the combined stress). Comparison of these responses are presented in Venn diagram (D). P, pathogen; D, drought; C, combined stress

## Slide 3
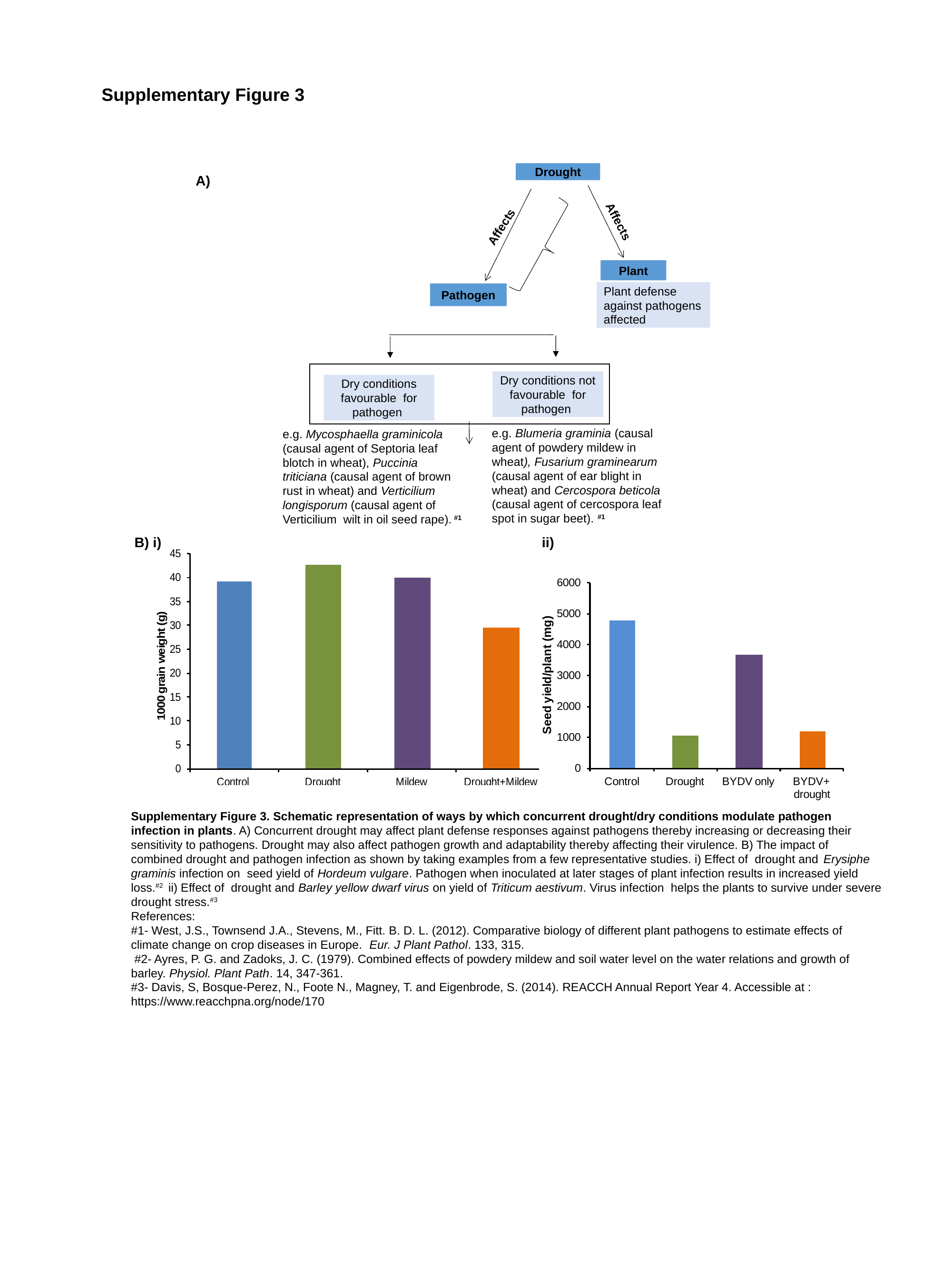

Supplementary Figure 3
Drought
Affects
Affects
Plant
Plant defense against pathogens affected
Pathogen
Dry conditions not favourable for pathogen
Dry conditions favourable for pathogen
e.g. Blumeria graminia (causal agent of powdery mildew in wheat), Fusarium graminearum (causal agent of ear blight in wheat) and Cercospora beticola (causal agent of cercospora leaf spot in sugar beet). #1
e.g. Mycosphaella graminicola (causal agent of Septoria leaf blotch in wheat), Puccinia triticiana (causal agent of brown rust in wheat) and Verticilium longisporum (causal agent of Verticilium wilt in oil seed rape). #1
A)
B) i)
ii)
Supplementary Figure 3. Schematic representation of ways by which concurrent drought/dry conditions modulate pathogen infection in plants. A) Concurrent drought may affect plant defense responses against pathogens thereby increasing or decreasing their sensitivity to pathogens. Drought may also affect pathogen growth and adaptability thereby affecting their virulence. B) The impact of combined drought and pathogen infection as shown by taking examples from a few representative studies. i) Effect of drought and Erysiphe graminis infection on seed yield of Hordeum vulgare. Pathogen when inoculated at later stages of plant infection results in increased yield loss.#2 ii) Effect of drought and Barley yellow dwarf virus on yield of Triticum aestivum. Virus infection helps the plants to survive under severe drought stress.#3
References:
#1- West, J.S., Townsend J.A., Stevens, M., Fitt. B. D. L. (2012). Comparative biology of different plant pathogens to estimate effects of climate change on crop diseases in Europe. Eur. J Plant Pathol. 133, 315.
 #2- Ayres, P. G. and Zadoks, J. C. (1979). Combined effects of powdery mildew and soil water level on the water relations and growth of barley. Physiol. Plant Path. 14, 347-361.
#3- Davis, S, Bosque-Perez, N., Foote N., Magney, T. and Eigenbrode, S. (2014). REACCH Annual Report Year 4. Accessible at : https://www.reacchpna.org/node/170
